# Mitochondrial DNA genomes revealed different patterns of high-altitude adaptation in high-altitude Tajiks compared with Tibetans and Sherpas

**DOI:** 10.1038/s41598-020-67519-z

**Published:** 2020-06-29

**Authors:** Yu Chen, Liang Gong, Xinyuan Liu, Xingshu Chen, Shenghong Yang, Yongjun Luo

**Affiliations:** 1 Department of Military Medical Geography, Army Health Service Training Base, Third Military Medical University (Army Medical University), Chongqing, 400038 China; 2 Health Department of the 957th Hospital of PLA, Ali, 859000 Tibet China

**Keywords:** Genetics research, Molecular medicine

## Abstract

High-altitude Tajiks (HA-Tajiks), Tibetans and Sherpas are three groups of high-altitude native people in China. The differences in the mtDNA genome between the three populations and the role of the mtDNA genome in the high-altitude adaptation of HA-Tajiks were seldom investigated. In this study, 80 HA-Tajiks were enrolled, and their whole mtDNA genomes were sequenced. The haplogroup of each subject was determined by comparison to the revised Cambridge Reference Sequence (rCRS). Ten additional populations from East Asia and Central Asia, including Tibetans and Sherpas, were selected as references. The top haplogroup was U, followed by H, T and J. Principle component analysis and genetic distance analysis indicated that HA-Tajiks showed a close relationship with Wakhi Tajiks, Pamiri Tajiks and Sarikoli Tajiks, indicating that they should be considered one nation scattered around the Pamirs. The difference in the mtDNA genome between HA-Tajiks and Sherpas was significantly greater than that between HA-Tajiks and Tibetans. Among the 13 genes related to the OXPHOS pathway encoded by the mtDNA genome, HA-Tajiks showed more significant differences in ND3 and CYTB compared to Tibetans. Compared to Sherpas, HA-Tajiks showed more significant differences in ND1, ND2, COX1, ATP8, ATP6, ND3, ND4L, ND4, ND5 and CYTB. The associated functional changes and underlying molecular mechanisms should be explored by molecular and biochemical investigations in further studies.

## Introduction

When humans spread throughout the Earth, high-altitude environments presented a significant challenge to the people living within them. Low air pressure, hypoxia, strong solar radiation and low temperature exert great pressure on the survival of local people. Genetic factors in both the nuclear genome and mitochondrial DNA (mtDNA) genome play great roles in the adaptation to high-altitude environments, which was considered as natural impacts on human evolution and adaptation^1^. In the past few years, an increasing number of genetic studies on the nuclear genome have shown that a series of genes have been involved in high-altitude adaptation in Tibetans^2–8^, Andeans^8,9^ and Ethiopians^10,11^, and these genes are mainly distributed in the hypoxia-inducible factor signaling pathway and the TP53 pathway. However, the role of the mtDNA genome in high-altitude adaptation are still in discussion and should receive more attention.

Mitochondria are known as the cell’s power plant, where cellular fuel is oxidized to provide energy for metabolism. Mitochondrial function is dependent on mtDNA, and mtDNA is well established as a genetic marker. A high mutation rate, small genome size, maternal inheritance, and lack of recombination make mtDNA an important tool for studying genetic structure in different populations. Besides, analyses of mtDNA sequences provide clues for exploring the genetic relationships between different populations, which contributes to deep understanding the role of mtDNA variations in human evolution. In addition to 2 rRNAs and 22 tRNAs, mtDNA encodes 13 core subunits related to oxidative phosphorylation (OXPHOS). Approximately 90% of the energy required by the cell is provided by OXPHOS, which is significantly affected by mtDNA variations. Hence, it is commonly believed that mtDNA contributed to high-altitude adaptation in high-altitude natives^12,13^.

There are three groups of high-altitude natives living in China, including high-altitude Tajiks (hereafter referred to as HA-Tajiks), Tibetans and Sherpas. The HA-Tajik population is one of 56 ethnic groups in China. HA-Tajiks have lived for generations in the Xinjiang Taxkorgan Tajik Autonomous County, where the average elevation is more than 4,000 m. Taxkorgan is perched in the highest part of the Pamirs. The world’s second highest peak, Mount Qogir, towers over the south, and in the north stands Mount Muztagata, “the father of ice peaks.” The HA-Tajik people seldom intermarry with other ethnic groups, and this ethnic identity means that their genetic structure shows little mixing with outsiders. Thus, it is attractive and feasible to explore the role of mtDNA in the high-altitude adaptation of HA-Tajiks. Tibetans are considered to be an ethnic group that is adapted to the high-altitude environment, and it has been reported that mtDNA variations influence the efficiency of oxygen utilization and function in native Tibetans^14^. Sherpas live south of the Himalayas and are famous for their physical ability in climbing Mount Everest and are well-known as porters in the Himalayas. Their characteristics at high altitudes are considered as markers of high-altitude adaptation. Previous studies have found that eNOS, PPARA^15^, HIF, ACE^16^ and EPAS1 in the nuclear genome and CYTB, ATP6, ND1, ND4 and ND5 in the mtDNA genome play a great role in the high-altitude adaptation of Sherpas^17^.

Recently, a study analyzed mtDNA genomes in different populations in Central Asia located around the Pamirs^18^, and different populations of Tajiks living at high altitude were also investigated. Because partial mtDNA sequences are not able to provide enough information, so complete mtDNA genome sequences were required for this research. To better understand the genetic structure of the mtDNA genome and identify the possible role of the mtDNA genome in high-altitude adaptation in HA-Tajiks, 80 HA-Tajik individuals living in Taxkorgan were enrolled, and their whole mtDNA genomes were sequenced. We also examined the mtDNA genomes of Tibetans and Sherpas as well as other reported Tajik populations^18^ to compare the genetic differences between them and to analyze different patterns of high-altitude adaptation between three high-altitude native populations at the Qinghai-Tibetan Plateau and in the Pamirs from perspectives of mtDNA variations.

## Results

### Study population and reference population in this study

A total of 11 populations, including 706 subjects, were analyzed in this study. The basic information of the subjects is listed in Table 1.

**Table 1:** Basic information of the populations enrolled in this study.

Population	Category	Size	Location	Source
High-altitude Tajik	Highlander	80	Taxkorgan, Xinjiang, China	This study
Sarikoli Tajik	Highlander	86	Taxkorgan, Xinjiang, China	Reference
Wakhi Tajik	Highlander	66	Taxkorgan, Xinjiang, China	Reference
Pamirs Tajik	Highlander	50	Gorno-Badakhshan, Tajikistan	Reference
Lowland Tajik	Lowlander	28	Dushanbe, Tajikistan	Reference
East Pamir Kyrgyz	Highlander	68	Taxkorgan, Xinjiang, China	Reference
Lowland Kyrgyz	Lowlander	54	Artux, Xinjiang, China	Reference
Lowland Uygur	Lowlander	27	Artux, Xinjiang, China	Reference
Sherpa	Highlander	76	Tibet, China	Reference
Beijing Han Chinese	Lowlander	103	Beijing, China	1000 Genomes Project
Tibetan	Highlander	68	Tibet, China	Reference

### mtDNA genetic diversity of Tajiks

**Figure 1:**
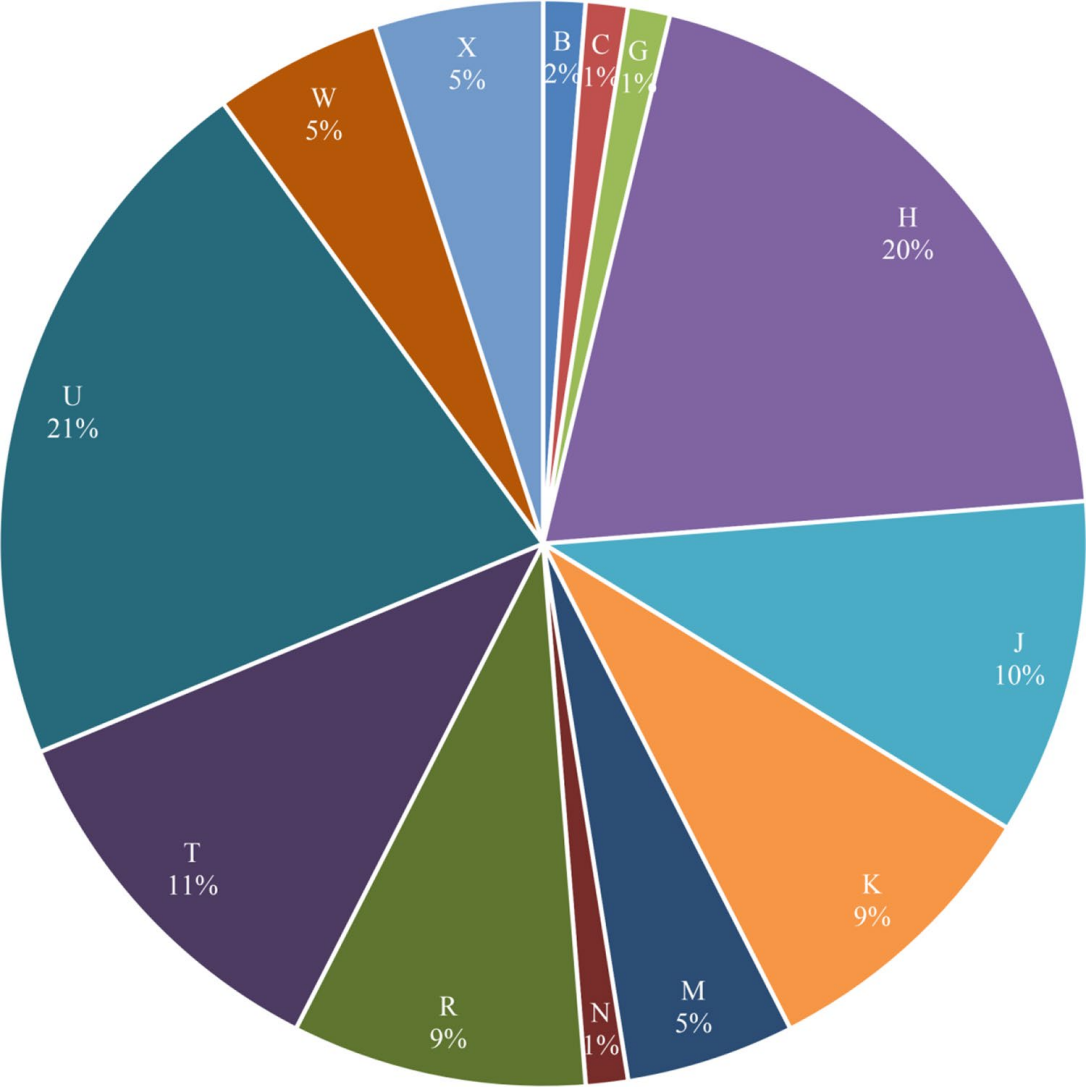
The haplogroup profiles of HA-Tajiks enrolled in this study. Major haplogroups were U, followed by H, T and J. Haplogroups were merged for brevity. Detailed frequencies of haplogroups in HA-Tajiks were listed in Table S2.

The number of haplotypes, nucleotide diversity, haplotype diversity, Tajima’s D and Fu’s Fs were 73, 0.00216 ± 0.00024, 0.997 ± 0.00001, − 2.277 and − 33.741, respectively. The main frequencies of the haplogroups in the 80 HA-Tajiks analyzed in this study are shown in Fig. 1, and the major haplogroups were U, followed by H, T and J, indicating that the HA-Tajik population settled in Taxkorgan, China, may have originated from Europe^18,19^. Haplogroup of each subject was provided in Table S1. Phylogeny of 80 HA-Tajiks calculated by mtPhyl (version 5.003) was provided as dataset1 with a xlsx file. Detailed frequencies of haplogroups in 80 HA-Tajiks as well as other reference population were provided in Table S2.

### Bayesian skyline plot (BSP) for HA-Tajiks

**Figure 2:**
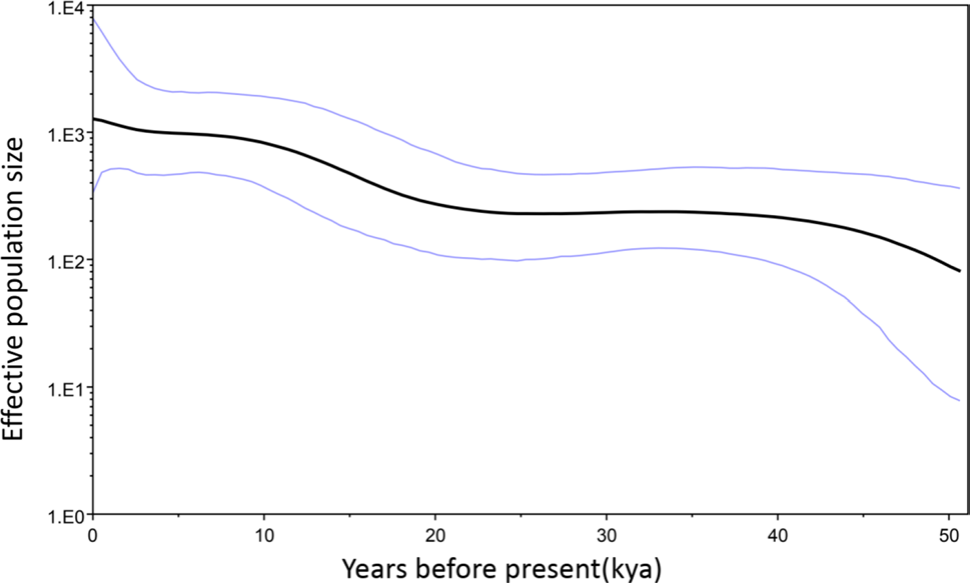
Bayesian Skyline Plot of HA-Tajik population history. BSP obtained by BEAST showed population history predicted from mitochondrial coding regions of 80 HA-Tajik subjects. The black line in the middle indicates the median population size predict from Bayesian posterior distribution. The population expansion may start from 10–15 kya before present.

BSP was conducted to trace historical variations in population size based on coding regions of mtDNA genome (Fig. 2). Although the sample size of HA-Tajiks is small in this study, it could be inferred that the effective population size of HA-Tajiks is steadily growing, which is consistent with demographic data of HA-Tajiks in China, especially in recent years (https://www.china.org.cn/e-groups/shaoshu/shao-2-tajik.htm). Besides, the expansion of HA-Tajiks revealed by the BSP is also in accordance with the negative Tajima’s D and Fu’s Fs.

### Genetic relationship of HA-Tajiks with other populations

**Figure 3:**
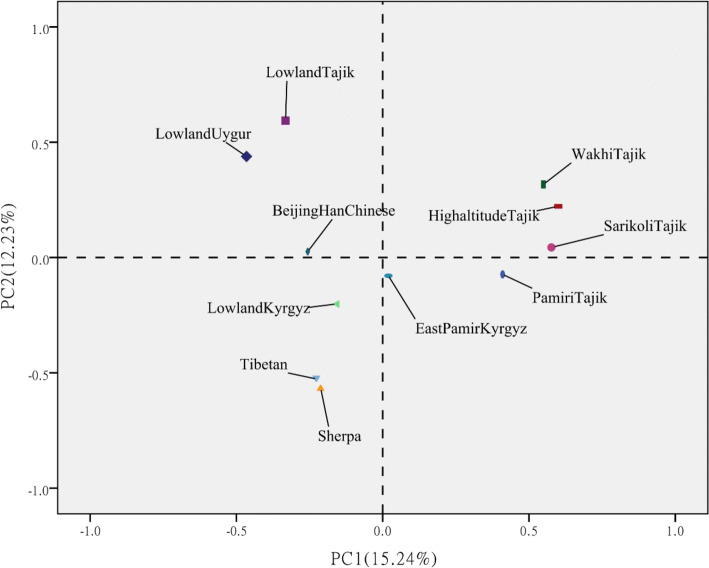
PCA of populations from HA-Tajiks and other ten populations. PC map of populations based on mtDNA haplogroup frequencies (Table S2).

**Figure 4:**
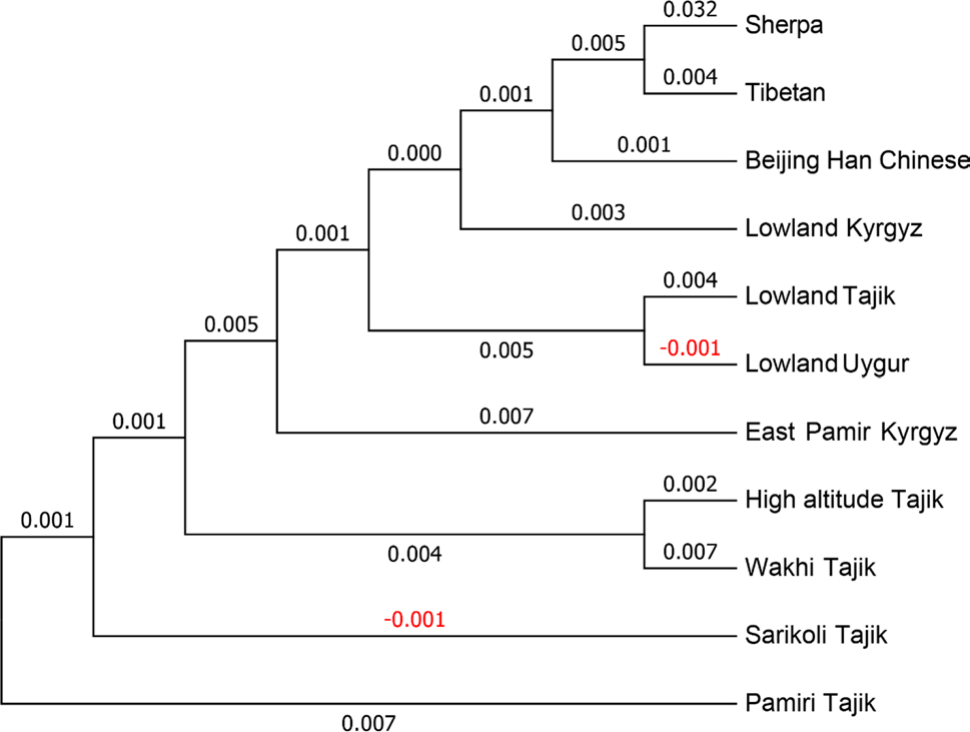
Genetic distance of populations from HA-Tajiks and other ten populations based on mtDNA haplogroup frequencies.

PCA was applied based on the frequencies of haplogroups in the mtDNA genomes to represent the relationships among the 11 populations enrolled in this study after transformation (Fig. 3). The detailed frequencies of the mtDNA haplogroups in the 11 populations are provided in Table S2. Further analysis based on ARLEQUIN 3.5.1.3 and MEGA 7.0 also showed that the HA-Tajiks enrolled in this study presented a close genetic relationship with the Wakhi Tajiks, Pamiri Tajiks and Sarikoli Tajiks (Figs. 3, 4), indicating that they may belong to one nation, with differences in their geographical distributions. Moreover, Figs. 3 and 4 indicate that the genetic relationships of HA-Tajiks with Tibetans and Sherpas are distant, while Tibetans and Sherpas show a close relationship. Hence, it could be inferred that HA-Tajiks may exhibit a different pattern of high-altitude adaptation compared to Tibetans and Sherpas from the perspective of the mtDNA genome. To further explore these differences, a detailed comparison of different regions in the mtDNA genome between HA-Tajiks and Tibetans as well as Sherpas was performed in subsequent analysis.

### Different variations in the mtDNA genome between Tajiks, Tibetans and Sherpas

After comparison to the rCRS, the number of variations in each polymorphism of the mtDNA genome was calculated in HA-Tajiks, Tibetans and Sherpas. Taking polymorphism 10400T of the mtDNA genome as an example, the numbers of variants were determined to be 6, 53 and 49, so the numbers of non-variants were 74, 15 and 27 in HA-Tajiks, Tibetans and Sherpas, respectively. At the statistical significance level of 0.025, our results indicated that there were significant differences in the distribution of many polymorphisms in the mtDNA genomes. As a result, only p values below 0.0000001 and polymorphisms in 13 genes belonging to the OXPHOS pathway encoded by the mtDNA genome are presented. The comparisons between HA-Tajiks and Tibetans and between HA-Tajiks and Sherpas are listed in Tables 2 and 3, and the remaining results related to OXPHOS are provided in Table S3 and Table S4.

**Table 2:** Results with p values below 0.0000001 in the comparison of the mtDNA genome between Tibetans and HA-Tajiks.

Polymorphisms	Amino acid change	Regions	Tibetan		HA-Tajiks		χ^2^	p values
			Variant	Non-variant	Variant	Non-variant		
10400 T	No	ND3	53	15	6	74	76.08	< 0.0000001
14783C	No	CYTB	53	15	6	74	76.08	< 0.0000001
15043A	No	CYTB	53	15	7	73	73.00	< 0.0000001
15301A	No	CYTB	53	15	6	74	76.08	< 0.0000001

**Table 3:** Results with p values below 0.0000001 in the comparison of the mtDNA genome between Sherpas and HA-Tajiks.

Polymorphisms	Amino acid change	Regions	Sherpa		HA-Tajiks		χ^2^	p values
			variant	non-variant	variant	non-variant		
3594T	No	ND1	0	76	40	40	51.10	< 0.0000001
4104G	No	ND1	0	76	40	40	51.10	< 0.0000001
4769G	No	ND2	76	0	42	38	47.73	< 0.0000001
7028T	No	COX1	75	1	43	37	42.71	< 0.0000001
7146G	Yes	COX1	0	76	40	40	51.10	< 0.0000001
7256T	No	COX1	3	73	40	40	41.40	< 0.0000001
8468T	No	ATP8	0	76	40	40	51.10	< 0.0000001
8655T	No	ATP6	0	76	40	40	51.10	< 0.0000001
8860G	Yes	ATP6	76	0	40	40	51.10	< 0.0000001
10400T	No	ND3	49	27	6	74	55.42	< 0.0000001
10664T	No	ND4L	0	76	40	40	51.10	< 0.0000001
10688A	No	ND4L	0	76	40	40	51.10	< 0.0000001
10810C	No	ND4	0	76	40	40	51.10	< 0.0000001
10915C	No	ND4	0	76	40	40	51.10	< 0.0000001
11719A	No	ND4	75	1	43	37	42.71	< 0.0000001
13105G	Yes	ND5	0	76	40	40	51.10	< 0.0000001
13276G	Yes	ND5	0	76	40	40	51.10	< 0.0000001
13506T	No	ND5	0	76	40	40	51.10	< 0.0000001
13650T	No	ND5	0	76	40	40	51.10	< 0.0000001
14766T	Yes	CYTB	75	1	43	37	42.71	< 0.0000001
14783C	No	CYTB	49	27	6	74	55.42	< 0.0000001
15043A	No	CYTB	49	27	7	73	52.59	< 0.0000001
15301A	No	CYTB	49	27	6	74	55.42	< 0.0000001
15326G	Yes	CYTB	76	0	40	40	51.10	< 0.0000001

Table 2 showed that the frequencies of the variant polymorphisms 10400T, 14783C, 15043A and 15301A in Tibetans were significantly higher than those in Tajiks. Polymorphism 10400T belongs to ND3 and polymorphisms 14783C, 15043A and 15301A to CYTB. Hence, it seemed that polymorphisms in CYTB may provide more clues about high-altitude adaptation in Tibetans on the Qinghai-Tibetan Plateau than in HA-Tajiks in the Pamirs, and other regions of the mtDNA genome should be evaluated in relation to high-altitude adaptation in HA-Tajiks.

Compared to Sherpas, HA-Tajiks exhibited more polymorphisms in multiple regions of the mtDNA genome, and detailed results are listed in Table 3. The mtDNA genome of HA-Tajiks showed significant differences in ND1, ND2, COX1, ATP8, ATP6, ND3, ND4L, ND4, ND5 and CYTB, which are more complicated than the comparisons between Tajiks and Tibetans. Polymorphisms 14783C and 15301A of CYTB showed the most significant differences in the distribution between HA-Tajiks and Sherpas. In addition, it could also be inferred that Tibetans and Sherpas may present different patterns of high-altitude adaptation from the perspective of the mtDNA genome, because frequencies of certain variants in mtDNA genome also showed significant differences between Tibetans and Sherpas.

## Discussion

This is the first study to compare and analyze molecular evidence of high-altitude adaptation in three high-altitude native groups from the perspective of the mitochondrial genome. In this study, the whole mtDNA genomes of 80 HA-Tajiks living in the Pamirs were sequenced, and the major mitochondrial haplogroups in HA-Tajiks were U, H and T as well as J confirmed that HA-Tajiks settled in the Pamirs were likely to be originated in Europe. BSP revealed by BEAST indicated that the effective population size was steadily increasing, which was in consistent with the current status of HA-Tajiks in China. Based on PCA and genetic distance analysis, we found that the HA-Tajiks enrolled in this study exhibited a close relationship with Wakhi Tajiks, Pamiri Tajiks and Sarikoli Tajiks. The difference in the mtDNA genome between HA-Tajiks and Sherpas was significantly greater than the differences between HA-Tajiks and Tibetans, suggesting different patterns of high-altitude adaptation were existed between HA-Tajiks, Tibetans and Sherpas.

BSP was an effective tool to analyze population size based on mtDNA genome sequences. However, different settings of analysis methods would generate various results even dealing with the same sequences. Compared to Peng’s research^18^, significant decline in effective population size was observed by BSPs, which is conflict with negative Tajima’s D and Fu’s Fs yielded in his research. Possible explanations were different sample strategies, and mainly were attributed to different parameters of strict clock model and analysis methods. In Peng’s research, this strict clock model was 1.404 × 10^−8^ substitutions per site per year and the TrN + I + G substitution model, and 2.308 × 10^−8^ substitutions per site per year as well as the GTR model of nucleotide substitution with empirical base frequencies were employed in this research. In addition, appropriate analytical methods are not easily determined when facing big data. Hence, parameters setting of software are not only depend on references but also in accordance to the actual situation as well as population history.

Although HA-Tajiks, Tibetans and Sherpas are high-altitude natives, they exhibited great differences in their languages, customs and origins^18,20^. Even after the long-term settlement of high-altitude environments, these three groups may show different responses to hypoxia stimulation at the molecular level, and variants in the mtDNA genome provided an useful tool for further analysis^1^. Mitochondrion have been reported to be involved in high-altitude adaptation in Tibetans and Sherpas. The mitochondrial nt3010G-nt3970C haplotype^21^ and haplogroup M9a1a1c1b in Tibetans^13,22^ and haplogroups C4a3b1 and A4e3a in Sherpas^17^ contributed to high-altitude adaptation. Compared to Tibetans, the distribution of polymorphisms 10400T, 14783C, 15043A and 15301A showed the most significant differences in Tajiks. However, the frequencies of these polymorphisms in Tajiks were significantly lower than those in Tibetans, indicating that other regions of the mtDNA genome should be considered in the assessment of the role of mtDNA in high-altitude adaptation in HA-Tajiks. Among the 13 genes belonging to the OXPHOS pathway, we found that the frequency of variant polymorphism 12372 (12372A) in ND5 was the most significantly increased compared to that in Tibetans (Table S3). Taking U haplogroup as an example, 12372A is the marker of the U haplogroup, which accounted for the main lineage in the 80 HA-Tajiks enrolled in this study. Functional changes in 12372A were not detected, and this polymorphism showed no association with sudden infant death syndrome in the Swiss population^23^. Although the 12372A variant in ND5 does not result in an amino acid change, it may affect the DNA-RNA network, mRNA stability, RNA splicing, translation kinetics and protein folding, etc.^15,24^, which ultimately contributes to disease progression. It seems that many pathogenic mutations are enriched in the mtDNA genomes of high-altitude native peoples^13^. However, these deleterious mutations do not induce a harmful phenotype in high-altitude natives, indicating that the diseases caused by mitochondrial mutations may be population specific. Hence, the role of 12372A in high-altitude adaptation in Tajiks needs to be reevaluated with larger samples, and the molecular mechanism associated with the 12372A mutation should be analyzed in further studies.

In the comparison between Tajiks and Sherpas, the frequencies of polymorphisms 3594T and 4104G in ND1, 7146G and 7256T in COX1, 8468T in ATP8, 8655T in ATP6, 10664T and 10688A in ND4L, 10810C and 10915C in ND4, and 13105G, 13276G, 13506T and 13650T in ND5 were found to be significantly higher in Tajiks. In addition, these variants seemed to be in linkage disequilibrium. Except for polymorphisms 7146G, 13105G and 13276G, all other polymorphisms mentioned above did not induce amino acid changes. 7146G, 13105G and 13276G result in amino acid changes from threonine to alanine, isoleucine to valine and methionine to valine, respectively. However, genotype–phenotype association and functional analyses based on three nonsynonymous amino acid mutations have seldom been reported. The 7146G mutation was located in COX1, and this variant may influence the biogenesis of COX1 and ultimately cause a different response at the molecular level in Tajiks in the Pamirs compared with Sherpas on the Qinghai-Tibetan plateau, which needs to be checked through functional analysis. 13105G and 13276G in ND5 were found to be associated with a risk of cervical cancer^25^. ND5 is a primary subunit of nicotinamide adenine dinucleotides, and a mutation in ND5 can induce higher levels of reactive oxygen species^26^. Combined with 13506T and 13650T in ND5, it seemed that alterations in ND5 would induce cumulative effects and may play a great role in high-altitude adaptation in Tajiks. However, the functional significance and the underlying mechanisms are unknown. Further investigations including molecular and biochemical studies are needed to explore the role of mutations in ND5 in high-altitude adaptation in HA-Tajiks.

With the development of DNA sequencing technologies, next-generation sequencing has been widely applied in genetic studies, and the accuracy has increased significantly. Traditional Sanger sequencing based on PCR products was performed in this study, and we found that checking DNA sequencing results and the assembly of DNA fragments manually is highly time and energy consuming. Hence, in the acquisition of whole mtDNA genomes, next-generation sequencing should be considered as the main tool in further analysis, and Sanger sequencing could be a powerful additional complement, especially when the results acquired by next-generation sequencing are ambiguous.

## Conclusion

We found that the lineages including U, H, T and J accounted for most HA-Tajik samples, indicating an European origin, and the HA-Tajik population showed a closer relationship with Wakhi Tajiks. BSP revealed by BEAST indicated that the effective population size was steadily increasing. Among 13 genes belonging to the OXPHOS pathway encoded by the mtDNA genome, HA-Tajiks showed the most significant differences in ND3 and CYTB compared to Tibetans. Compared to Sherpas, HA-Tajiks showed the most significant differences in ND1, ND2, COX1, ATP8, ATP6, ND3, ND4L, ND4, ND5 and CYTB. The associated biochemical changes and molecular mechanisms should be explored by functional investigations in further studies.

## Materials and methods

### Ethics statements

This research was approved by the ethics committee of the Third Military Medical University and the 950th Hospital of the PLA of China. All experiments were performed in accordance with relevant guidelines and regulations. All participants provided written informed consent before this investigation commenced.

### Study population

A total of 80 unrelated HA-Tajiks (19 males, 52.9 ± 14.9 years, 19–76 years old; 61 females, 46.0 ± 15.0 years, 25–83 years old) were enrolled in this study. All subjects confirmed that they and their parents had been born and lived in Taxkorgan their whole lives in a questionnaire. Genomic DNA was extracted from venous blood using a DNA isolation kit (Omega Bio-Tek, Inc., Norcross, GA, USA) and was stored at − 20 °C for further use.

### mtDNA genome amplification and sequencing

Eight pairs of primers were applied to amplify the mtDNA genome by polymerase chain reaction (PCR), and 22 primers were used for sequencing the PCR products^26^ by Sanger dideoxy sequencing (detailed information of PCR amplification and sequencing primers is listed in Table S5 and S6). The sequence of every PCR product was carefully checked according to the revised Cambridge Reference Sequence (rCRS) (GenBank: NC_012920). All of the sequences of the PCR products from each subject were integrated, and overlapping sequences were removed. After manual check of sequence diagrams, we found that the sequencing accuracy of 315–598 in sample 106, 120, 122, 133, 148, 149, 156, 167, 304, 320, and 16190–16396 in sample 119, 138, 225 were not satisfactory because of poly C structure existed. To make our work more convincing, we delete the mtDNA sequences in 315–598 and 16190–16396 belonging to D-loop region in samples mentioned above respectively, and these sequences were replaced as sequencing gaps. Finally, we obtained the mitochondrial genome sequence of 80 HA-Tajiks without 315–598 or 16189–16396 belonging to D-loop region for the following analysis.

### mtDNA haplogroup analysis and reference mtDNA genomes

Each mtDNA genome of 80 HA-Tajik individuals was compared to Phylotree 17 rCRS to confirm variant sites and haplogroup by using Mitotool (https://mitotool.kiz.ac.cn/)^27^ Ten additional populations living in East Asia and Central Asia, including Tibetans and Sherpas, were selected as references. The GenBank identification numbers of the Tibetans and Sherpas are provided as supplementary materials in Table S7. Sequence data for the mtDNA genome were acquired from the 1000 Genomes Project or downloaded from GenBank as the references indicate. Variant sites and haplogroups were also acquired by using Mitotool as mentioned above. All the mtDNA sequences of 80 HA-Tajiks as mentioned above were deposited in Genbank (MT554196-MT554275).

### Analysis of mtDNA genomes

The mtDNA genetic diversity of HA-Tajiks (including haplotype numbers, nucleotide and haplotype diversity, Tajima’s D, and Fu’s Fs) was calculated by DnaSP6.0^28^. The genetic distances between the HA-Tajiks and the reference populations based on the mtDNA genomes were analyzed by PCA based on the haplogroup distributions in different populations after normalized transformation^29^. The Fst values between the populations enrolled in this study were calculated with ARLEQUIN 3.5.1.3^30^. A phylogenetic tree was constructed with a neighbor-joining tree with MEGA 7.0. The phylogeny was constructed by using mtPhyl^31^ (https://sites.google.com/site/mtphyl/home) with 80 complete mtDNA sequences in HA-Tajiks. The variants located in regions 16519, 16180–16193 and 310–315 and the AC indels in regions 515–522 were not included in the subsequent analysis^18^.

### Bayesian phylogenetic analysis of HA-Tajiks

The Bayesian skyline plots (BSPs) for HA-Tajiks were performed by BEAST v1.7.5. mtDNA coding region (np 577–16024) of each HA-Tajik subject was acquired by comparison of rCRS. After alignment of 80 mitochondrial coding region sequences, we employed the GTR model of nucleotide substitution with empirical base frequencies, and selected a strict clock model with the rate of 2.038 × 10^–8^ subs/site/year^32^. Markov chain Monte Carlo (MVMV) was applied to estimate coalescent history. Parameters of MVMV were set as running for 400,000,000 generations and calculated every 4,000 generations. Results yielded by BEAST were visualized by Tracer 1.5 (https://tree.bio.ed.ac.uk/software/tracer/).

### Detailed differences in the mtDNA genomes between Tajiks, Tibetans and Sherpas

To further identify the differences between HA-Tajiks, Tibetans and Sherpas, their whole-mtDNA-genome sequences were compared to the rCRS to confirm the variants in the mtDNA genomes in detail. The number of variants in different regions of the mtDNA genome was calculated by direct counting, and the variant distributions in different regions were analyzed by the chi-square test, which may reveal different patterns of high-altitude adaptation among HA-Tajiks, Tibetans and Sherpas. For the chi-square test, a Bonferroni correction was applied for multiple tests. Because the number of comparisons was 2 (HA-Tajik vs Tibetan, HA-Tajik vs Sherpa), the statistical significance level was set at 0.05/2 (0.025), and all p values were two-tailed.

## Supplementary information

Supplementary Table 1.

Supplementary Table 2.

Supplementary Table 3.

Supplementary Table 4.

Supplementary Table 5.

Supplementary Table 6.

Supplementary Table 7.

Supplementary information.

## Data Availability

The authors used mitochondrial sequence data in Genbank, all the sequence data could be downloaded as Genbank number indicating from National Center of Biotechnology Information. All other relevant data are provided in the paper and Supporting files.
