# Supplementary material for: Mitochondrial DNA genomes revealed different patterns of high-altitude adaptation in high-altitude Tajiks compared with Tibetans and Sherpas
Source: Sci Rep. 2020 Jun 29;10:10592. doi: 10.1038/s41598-020-67519-z (PMC7324373; doi:10.1038/s41598-020-67519-z)
Supplement: Supplementary file 1 — Supplementary Table 1. [file 41598_2020_67519_MOESM1_ESM.pdf]

Mitochondrial DNA genomes revealed different patterns of high-altitude adaptation in high-altitude Tajiks compared with Tibetans and Sherpas

Yu Chen[1], Liang Gong[1], Xinyuan Liu[1], Xingshu Chen[1], Shenghong Yang[2#], Yongjun Luo[1#]

(1 Department of Military Medical Geography, Army Health Service Training Base, Third Military Medical University(Army Medical University), Chongqing 40038, China; 2 Health Department of the 957th Hospital of PLA, Ali, Tibet, China 859000)

[#]Corresponding author:

Prof. Shenghong Yang, Health Department of the 957th Hospital of PLA, Ali, Tibet, 859000, China.

(E-mail: kevinysh0751@163.com)

Prof. Yongjun Luo, Army Health Service Training Base, Third Military Medical University(Army Medical University), Chongqing 400038, China.

(E-mail: luo.yongjun@qq.com)

Table S1 Haplogroup of each subject in 80 HA-Tajiks

Sample number	Haplogroup
101	J1b6
102	H6b2
104	R0a
105	H5b
106	H6b2
107	K1a12a
108	U5a2b
109	H20
110	H14a
111	R2
112	M3a1b
113	R2
114	C4a1
115	U5a1a1
116	U5a2b
117	T1a1n
118	T2d1b
119	B4c1a2
120	H6b2
121	T1a1n
122	H5b
123	U2b2
126	J1b1a1
129	J2b1a2a
130	H14a
131	U4b2
132	T
133	R0a'b
135	R2
136	K1b2a
137	T1a1n
138	X2c
139	U4b2
140	K1a12
141	K1b2a
143	U2b2
144	T2d1b
146	W3
148	H15a1b
149	H14a

150	U4b2
151	T2d1b
152	T2
153	K1a12
154	H2a1a
155	T2d1b
156	U4b2
158	W3b
159	H15a1a1
162	R2
166	H5b
167	R0a
171	J1d1
188	U4b2
190	H6b2
194	U4b2
203	J1b1a1
204	U4b2
212	J1b1a1
216	W3b
217	K1a12a
219	H14a
220	U5a2a1
221	M3a1b
225	X2c
230	U2b2
236	M7c1
242	M3a1b
300	K1b2a
304	X2
306	J1b1a
307	U4b2
312	U4b1a1a1
320	N1a1b1
321	G3a1
324	U4b1a1a1
329	H31b
331	X2
343	W1c
346	J1b1a1
