# Supplementary material for: Mitochondrial DNA genomes revealed different patterns of high-altitude adaptation in high-altitude Tajiks compared with Tibetans and Sherpas
Source: Sci Rep. 2020 Jun 29;10:10592. doi: 10.1038/s41598-020-67519-z (PMC7324373; doi:10.1038/s41598-020-67519-z)
Supplement: Supplementary file 2 — Supplementary Table 2. [file 41598_2020_67519_MOESM2_ESM.pdf]

# Mitochondrial DNA genomes revealed different patterns of high-altitude adaptation in high-altitude Tajiks compared with Tibetans and Sherpas

Yu Chen[1], Liang Gong[1], Xinyuan Liu[1], Xingshu Chen[1], Shenghong Yang[2#], Yongjun Luo[1#]

(1 Department of Military Medical Geography, Army Health Service Training Base, Third Military Medical University(Army Medical University), Chongqing 40038, China; 2 Health Department of the 957th Hospital of PLA, Ali, Tibet, China 859000)

[#]Corresponding author:

Prof. Shenghong Yang, Health Department of the 957th Hospital of PLA, Ali, Tibet, 859000, China.

(E-mail: kevinysh0751@163.com)

Prof. Yongjun Luo, Army Health Service Training Base, Third Military Medical University(Army Medical University), Chongqing 400038, China.

(E-mail: luo.yongjun@qq.com)

Table S2 Frequencies of haplogroups in different populations enrolled in this study

| Haplogroups | CHB | East Pamir Kyrgyz | High altitude Tajik | Lowland Kyrgyz | Lowland Tajik | Lowland Uygur | Pamiri Tajik | Sarikoli Tajik | Sherpa | Tibetan | Wakhi Tajik |
|---|---|---|---|---|---|---|---|---|---|---|---|
| A | 0.00 | 0.00 | 0.00 | 0.00 | 0.00 | 7.41 | 0.00 | 0.00 | 0.00 | 0.00 | 0.00 |
| A1 | 1.94 | 0.00 | 0.00 | 0.00 | 0.00 | 0.00 | 0.00 | 0.00 | 0.00 | 0.00 | 0.00 |
| A11a | 0.00 | 0.00 | 0.00 | 0.00 | 0.00 | 0.00 | 0.00 | 0.00 | 0.00 | 5.88 | 0.00 |
| A11a1a | 0.00 | 0.00 | 0.00 | 0.00 | 0.00 | 0.00 | 0.00 | 0.00 | 2.63 | 0.00 | 0.00 |
| A11b | 0.00 | 0.00 | 0.00 | 0.00 | 0.00 | 0.00 | 0.00 | 0.00 | 0.00 | 1.47 | 0.00 |
| A14 | 0.00 | 0.00 | 0.00 | 1.85 | 0.00 | 0.00 | 0.00 | 0.00 | 0.00 | 0.00 | 0.00 |
| A15 | 0.97 | 0.00 | 0.00 | 0.00 | 0.00 | 0.00 | 0.00 | 0.00 | 0.00 | 0.00 | 0.00 |
| A15a | 0.00 | 0.00 | 0.00 | 0.00 | 0.00 | 0.00 | 0.00 | 0.00 | 0.00 | 1.47 | 0.00 |
| A15c1 | 0.00 | 0.00 | 0.00 | 0.00 | 0.00 | 0.00 | 0.00 | 0.00 | 18.42 | 0.00 | 0.00 |
| A17 | 0.97 | 1.47 | 0.00 | 0.00 | 0.00 | 0.00 | 0.00 | 0.00 | 0.00 | 0.00 | 0.00 |
| A18 | 0.97 | 0.00 | 0.00 | 0.00 | 0.00 | 0.00 | 0.00 | 0.00 | 0.00 | 0.00 | 0.00 |
| A19 | 0.97 | 0.00 | 0.00 | 0.00 | 0.00 | 0.00 | 0.00 | 0.00 | 0.00 | 0.00 | 0.00 |
| A1a | 0.00 | 0.00 | 0.00 | 0.00 | 0.00 | 0.00 | 0.00 | 1.16 | 0.00 | 0.00 | 0.00 |
| A21 | 0.00 | 0.00 | 0.00 | 0.00 | 0.00 | 0.00 | 0.00 | 0.00 | 2.63 | 0.00 | 0.00 |
| A5b1 | 0.97 | 0.00 | 0.00 | 0.00 | 0.00 | 0.00 | 0.00 | 0.00 | 0.00 | 0.00 | 0.00 |
| A6b | 0.00 | 0.00 | 0.00 | 0.00 | 0.00 | 0.00 | 0.00 | 0.00 | 2.63 | 1.47 | 0.00 |
| B4a1 | 0.00 | 0.00 | 0.00 | 0.00 | 0.00 | 0.00 | 0.00 | 0.00 | 0.00 | 1.47 | 0.00 |
| B4a1c5 | 0.97 | 0.00 | 0.00 | 0.00 | 0.00 | 0.00 | 0.00 | 0.00 | 0.00 | 0.00 | 0.00 |
| B4a2b | 0.97 | 0.00 | 0.00 | 0.00 | 0.00 | 0.00 | 0.00 | 0.00 | 0.00 | 0.00 | 0.00 |
| B4a3 | 0.00 | 0.00 | 0.00 | 0.00 | 0.00 | 0.00 | 0.00 | 0.00 | 0.00 | 1.47 | 0.00 |
| B4a4 | 1.94 | 0.00 | 0.00 | 0.00 | 0.00 | 0.00 | 0.00 | 0.00 | 0.00 | 0.00 | 0.00 |
| B4a5 | 0.97 | 0.00 | 0.00 | 0.00 | 0.00 | 0.00 | 0.00 | 0.00 | 0.00 | 0.00 | 0.00 |
| B4b1a2a | 0.97 | 0.00 | 0.00 | 0.00 | 0.00 | 0.00 | 0.00 | 0.00 | 0.00 | 0.00 | 0.00 |
| B4c1a2 | 0.00 | 0.00 | 1.25 | 0.00 | 0.00 | 0.00 | 0.00 | 1.16 | 0.00 | 0.00 | 0.00 |
| B4c1a2a | 0.00 | 0.00 | 0.00 | 3.70 | 0.00 | 0.00 | 0.00 | 0.00 | 0.00 | 0.00 | 0.00 |
| B4c1b2c | 0.97 | 0.00 | 0.00 | 0.00 | 0.00 | 0.00 | 0.00 | 0.00 | 0.00 | 0.00 | 0.00 |
| B4c1b2c1 | 0.97 | 0.00 | 0.00 | 0.00 | 0.00 | 0.00 | 0.00 | 0.00 | 0.00 | 0.00 | 0.00 |
| B4d1 | 0.97 | 0.00 | 0.00 | 0.00 | 0.00 | 0.00 | 0.00 | 0.00 | 0.00 | 0.00 | 0.00 |
| B4d1a | 0.97 | 0.00 | 0.00 | 0.00 | 0.00 | 0.00 | 0.00 | 0.00 | 0.00 | 0.00 | 0.00 |
| B4d3 | 0.97 | 0.00 | 0.00 | 0.00 | 0.00 | 0.00 | 0.00 | 0.00 | 0.00 | 0.00 | 0.00 |
| B4h1 | 0.97 | 0.00 | 0.00 | 0.00 | 0.00 | 0.00 | 0.00 | 0.00 | 0.00 | 0.00 | 0.00 |

| | | | | | | | | | | | |
|---|---|---|---|---|---|---|---|---|---|---|---|
| B4i1 | 0.97 | 0.00 | 0.00 | 0.00 | 0.00 | 0.00 | 0.00 | 0.00 | 0.00 | 0.00 | 0.00 |
| B4k | 1.94 | 0.00 | 0.00 | 0.00 | 0.00 | 0.00 | 0.00 | 0.00 | 0.00 | 0.00 | 0.00 |
| B5b2b | 0.97 | 0.00 | 0.00 | 0.00 | 0.00 | 0.00 | 0.00 | 0.00 | 0.00 | 0.00 | 0.00 |
| B5b4 | 0.00 | 0.00 | 0.00 | 3.70 | 0.00 | 0.00 | 0.00 | 0.00 | 0.00 | 0.00 | 0.00 |
| C | 0.97 | 0.00 | 0.00 | 0.00 | 0.00 | 0.00 | 0.00 | 0.00 | 0.00 | 0.00 | 0.00 |
| C1f | 0.00 | 0.00 | 0.00 | 0.00 | 0.00 | 0.00 | 2.00 | 0.00 | 0.00 | 0.00 | 0.00 |
| C4 | 0.00 | 0.00 | 0.00 | 0.00 | 0.00 | 0.00 | 2.00 | 1.16 | 0.00 | 0.00 | 0.00 |
| C4a1 | 0.00 | 2.94 | 1.25 | 0.00 | 0.00 | 0.00 | 0.00 | 2.33 | 0.00 | 0.00 | 1.52 |
| C4a1a | 0.00 | 0.00 | 0.00 | 1.85 | 0.00 | 7.41 | 0.00 | 2.33 | 0.00 | 0.00 | 1.52 |
| C4a1a2 | 0.97 | 0.00 | 0.00 | 0.00 | 0.00 | 0.00 | 0.00 | 0.00 | 0.00 | 0.00 | 0.00 |
| C4a1a4a | 0.00 | 0.00 | 0.00 | 3.70 | 0.00 | 0.00 | 0.00 | 0.00 | 0.00 | 1.47 | 0.00 |
| C4a2b2 | 0.97 | 0.00 | 0.00 | 0.00 | 0.00 | 0.00 | 0.00 | 0.00 | 3.95 | 1.47 | 0.00 |
| C4a2b2a | 0.00 | 0.00 | 0.00 | 0.00 | 0.00 | 0.00 | 0.00 | 0.00 | 14.47 | 0.00 | 0.00 |
| C4a2c | 0.00 | 0.00 | 0.00 | 1.85 | 0.00 | 0.00 | 0.00 | 0.00 | 0.00 | 0.00 | 0.00 |
| C4a2c1 | 0.00 | 0.00 | 0.00 | 0.00 | 0.00 | 0.00 | 2.00 | 0.00 | 0.00 | 0.00 | 0.00 |
| C4b1 | 0.00 | 5.88 | 0.00 | 0.00 | 0.00 | 0.00 | 0.00 | 1.16 | 0.00 | 0.00 | 0.00 |
| C4d | 0.97 | 0.00 | 0.00 | 0.00 | 0.00 | 0.00 | 0.00 | 0.00 | 0.00 | 0.00 | 0.00 |
| C5b1a | 0.00 | 1.47 | 0.00 | 0.00 | 0.00 | 0.00 | 0.00 | 0.00 | 0.00 | 0.00 | 0.00 |
| C7a | 0.97 | 0.00 | 0.00 | 0.00 | 0.00 | 0.00 | 0.00 | 0.00 | 0.00 | 0.00 | 0.00 |
| D4 | 0.00 | 0.00 | 0.00 | 0.00 | 0.00 | 3.70 | 0.00 | 2.33 | 1.32 | 0.00 | 0.00 |
| D4a | 2.91 | 0.00 | 0.00 | 0.00 | 0.00 | 0.00 | 0.00 | 0.00 | 0.00 | 0.00 | 0.00 |
| D4a5 | 0.00 | 0.00 | 0.00 | 0.00 | 0.00 | 0.00 | 0.00 | 0.00 | 0.00 | 1.47 | 0.00 |
| D4a8 | 0.97 | 0.00 | 0.00 | 0.00 | 0.00 | 0.00 | 0.00 | 0.00 | 0.00 | 0.00 | 0.00 |
| D4b1a2a | 0.97 | 0.00 | 0.00 | 0.00 | 0.00 | 0.00 | 0.00 | 0.00 | 0.00 | 0.00 | 0.00 |
| D4b2b | 0.00 | 0.00 | 0.00 | 0.00 | 0.00 | 3.70 | 0.00 | 0.00 | 0.00 | 0.00 | 0.00 |
| D4b2b2b | 0.97 | 0.00 | 0.00 | 0.00 | 0.00 | 0.00 | 0.00 | 0.00 | 0.00 | 0.00 | 0.00 |
| D4b2b6 | 0.97 | 0.00 | 0.00 | 0.00 | 0.00 | 0.00 | 0.00 | 0.00 | 0.00 | 0.00 | 0.00 |
| D4c1a1 | 0.00 | 1.47 | 0.00 | 0.00 | 0.00 | 0.00 | 0.00 | 0.00 | 0.00 | 0.00 | 0.00 |
| D4c2 | 0.00 | 0.00 | 0.00 | 0.00 | 0.00 | 3.70 | 0.00 | 0.00 | 0.00 | 0.00 | 0.00 |
| D4e1a | 0.00 | 0.00 | 0.00 | 0.00 | 0.00 | 3.70 | 0.00 | 0.00 | 0.00 | 0.00 | 0.00 |
| D4e4a | 0.00 | 0.00 | 0.00 | 0.00 | 0.00 | 3.70 | 0.00 | 0.00 | 0.00 | 0.00 | 0.00 |
| D4e5a | 0.00 | 0.00 | 0.00 | 1.85 | 0.00 | 0.00 | 0.00 | 0.00 | 0.00 | 0.00 | 0.00 |
| D4e5b | 0.00 | 0.00 | 0.00 | 1.85 | 0.00 | 0.00 | 0.00 | 0.00 | 0.00 | 0.00 | 0.00 |
| D4g2a1 | 0.97 | 0.00 | 0.00 | 0.00 | 0.00 | 0.00 | 0.00 | 0.00 | 0.00 | 0.00 | 0.00 |

| | | | | | | | | | | | |
|---|---|---|---|---|---|---|---|---|---|---|---|
| D4h4a | 0.00 | 2.94 | 0.00 | 0.00 | 0.00 | 0.00 | 0.00 | 0.00 | 0.00 | 0.00 | 0.00 |
| D4i | 0.97 | 0.00 | 0.00 | 0.00 | 0.00 | 0.00 | 0.00 | 0.00 | 0.00 | 0.00 | 0.00 |
| D4j | 0.00 | 2.94 | 0.00 | 1.85 | 0.00 | 0.00 | 0.00 | 0.00 | 0.00 | 0.00 | 0.00 |
| D4j13 | 0.00 | 0.00 | 0.00 | 3.70 | 0.00 | 0.00 | 0.00 | 0.00 | 3.95 | 0.00 | 0.00 |
| D4j1a1 | 0.00 | 0.00 | 0.00 | 1.85 | 0.00 | 0.00 | 0.00 | 0.00 | 1.32 | 1.47 | 0.00 |
| D4j1b | 0.00 | 2.94 | 0.00 | 0.00 | 0.00 | 0.00 | 0.00 | 0.00 | 0.00 | 0.00 | 0.00 |
| D4j5a | 0.00 | 0.00 | 0.00 | 1.85 | 0.00 | 0.00 | 0.00 | 0.00 | 0.00 | 0.00 | 0.00 |
| D4j8 | 0.00 | 0.00 | 0.00 | 1.85 | 0.00 | 0.00 | 0.00 | 0.00 | 0.00 | 0.00 | 0.00 |
| D4k | 0.97 | 0.00 | 0.00 | 0.00 | 0.00 | 0.00 | 0.00 | 0.00 | 0.00 | 0.00 | 0.00 |
| D4l2b | 0.00 | 0.00 | 0.00 | 0.00 | 0.00 | 0.00 | 0.00 | 0.00 | 0.00 | 1.47 | 0.00 |
| D4m | 0.00 | 0.00 | 0.00 | 1.85 | 0.00 | 0.00 | 0.00 | 0.00 | 0.00 | 0.00 | 0.00 |
| D4m2a | 0.97 | 0.00 | 0.00 | 0.00 | 0.00 | 0.00 | 0.00 | 0.00 | 0.00 | 0.00 | 0.00 |
| D4n2 | 0.00 | 1.47 | 0.00 | 0.00 | 0.00 | 0.00 | 0.00 | 0.00 | 0.00 | 0.00 | 0.00 |
| D4o1 | 0.00 | 0.00 | 0.00 | 3.70 | 0.00 | 0.00 | 0.00 | 0.00 | 0.00 | 0.00 | 0.00 |
| D4s | 0.00 | 1.47 | 0.00 | 0.00 | 0.00 | 0.00 | 0.00 | 1.16 | 0.00 | 1.47 | 0.00 |
| D5a | 0.97 | 0.00 | 0.00 | 0.00 | 0.00 | 0.00 | 0.00 | 0.00 | 0.00 | 0.00 | 0.00 |
| D5a2a | 2.91 | 0.00 | 0.00 | 0.00 | 0.00 | 0.00 | 0.00 | 0.00 | 0.00 | 0.00 | 0.00 |
| D5a2a; D5a2a1 | 0.97 | 0.00 | 0.00 | 0.00 | 0.00 | 0.00 | 0.00 | 0.00 | 0.00 | 0.00 | 0.00 |
| D5a2a1 | 0.97 | 0.00 | 0.00 | 0.00 | 0.00 | 0.00 | 0.00 | 0.00 | 1.32 | 1.47 | 0.00 |
| D5a2a1a | 0.97 | 0.00 | 0.00 | 0.00 | 0.00 | 0.00 | 0.00 | 0.00 | 0.00 | 0.00 | 0.00 |
| D5a2a1b | 0.00 | 0.00 | 0.00 | 1.85 | 0.00 | 0.00 | 0.00 | 0.00 | 0.00 | 1.47 | 0.00 |
| D5a2a2 | 0.97 | 0.00 | 0.00 | 1.85 | 0.00 | 0.00 | 0.00 | 0.00 | 0.00 | 0.00 | 0.00 |
| D5a2b | 0.00 | 0.00 | 0.00 | 0.00 | 0.00 | 0.00 | 0.00 | 0.00 | 2.63 | 0.00 | 0.00 |
| D5b1b | 0.97 | 0.00 | 0.00 | 0.00 | 0.00 | 0.00 | 0.00 | 0.00 | 0.00 | 0.00 | 0.00 |
| D5b1c1; D5b1c1a | 0.97 | 0.00 | 0.00 | 0.00 | 0.00 | 0.00 | 0.00 | 0.00 | 0.00 | 0.00 | 0.00 |
| D5c | 0.97 | 0.00 | 0.00 | 0.00 | 0.00 | 0.00 | 0.00 | 0.00 | 0.00 | 0.00 | 0.00 |
| D5c1 | 0.97 | 0.00 | 0.00 | 0.00 | 0.00 | 0.00 | 0.00 | 0.00 | 0.00 | 0.00 | 0.00 |
| F1a1c | 0.97 | 0.00 | 0.00 | 0.00 | 0.00 | 0.00 | 0.00 | 0.00 | 0.00 | 0.00 | 0.00 |
| F1a1c3 | 0.97 | 0.00 | 0.00 | 0.00 | 0.00 | 0.00 | 0.00 | 0.00 | 0.00 | 0.00 | 0.00 |
| F1b1 | 0.97 | 0.00 | 0.00 | 0.00 | 0.00 | 3.70 | 0.00 | 0.00 | 0.00 | 1.47 | 0.00 |
| F1b1e | 0.00 | 0.00 | 0.00 | 1.85 | 0.00 | 0.00 | 0.00 | 0.00 | 0.00 | 0.00 | 0.00 |
| F1c1a1a | 0.00 | 0.00 | 0.00 | 0.00 | 0.00 | 0.00 | 0.00 | 0.00 | 1.32 | 1.47 | 0.00 |
| F1d | 1.94 | 0.00 | 0.00 | 1.85 | 0.00 | 0.00 | 0.00 | 0.00 | 0.00 | 1.47 | 0.00 |
| F1d1 | 0.97 | 0.00 | 0.00 | 0.00 | 0.00 | 0.00 | 0.00 | 0.00 | 0.00 | 0.00 | 0.00 |

| | | | | | | | | | | | |
|---|---|---|---|---|---|---|---|---|---|---|---|
| F1e2 | 0.97 | 1.47 | 0.00 | 0.00 | 0.00 | 0.00 | 0.00 | 0.00 | 0.00 | 0.00 | 0.00 |
| F1g | 0.00 | 1.47 | 0.00 | 0.00 | 0.00 | 0.00 | 0.00 | 0.00 | 0.00 | 0.00 | 0.00 |
| F2 | 0.00 | 0.00 | 0.00 | 0.00 | 0.00 | 3.70 | 0.00 | 0.00 | 0.00 | 0.00 | 0.00 |
| F2a | 0.97 | 0.00 | 0.00 | 0.00 | 0.00 | 0.00 | 0.00 | 0.00 | 0.00 | 0.00 | 0.00 |
| F2b1 | 1.94 | 0.00 | 0.00 | 0.00 | 0.00 | 0.00 | 0.00 | 0.00 | 0.00 | 0.00 | 0.00 |
| F2c2 | 0.97 | 0.00 | 0.00 | 0.00 | 0.00 | 0.00 | 0.00 | 0.00 | 0.00 | 0.00 | 0.00 |
| F2d | 1.94 | 0.00 | 0.00 | 0.00 | 0.00 | 0.00 | 0.00 | 0.00 | 0.00 | 0.00 | 0.00 |
| F2e | 0.97 | 0.00 | 0.00 | 0.00 | 0.00 | 0.00 | 0.00 | 0.00 | 0.00 | 0.00 | 0.00 |
| F2h | 0.00 | 0.00 | 0.00 | 0.00 | 0.00 | 0.00 | 0.00 | 0.00 | 0.00 | 1.47 | 0.00 |
| F2i | 0.97 | 0.00 | 0.00 | 0.00 | 0.00 | 0.00 | 0.00 | 0.00 | 0.00 | 0.00 | 0.00 |
| F3a1 | 0.00 | 2.94 | 0.00 | 0.00 | 0.00 | 0.00 | 0.00 | 0.00 | 0.00 | 0.00 | 0.00 |
| F4b | 0.97 | 0.00 | 0.00 | 0.00 | 0.00 | 0.00 | 0.00 | 0.00 | 0.00 | 0.00 | 0.00 |
| G1 | 0.97 | 0.00 | 0.00 | 0.00 | 0.00 | 0.00 | 0.00 | 0.00 | 0.00 | 0.00 | 0.00 |
| G1a1 | 0.00 | 0.00 | 0.00 | 0.00 | 0.00 | 0.00 | 4.00 | 3.49 | 0.00 | 0.00 | 0.00 |
| G1c2 | 0.97 | 0.00 | 0.00 | 0.00 | 0.00 | 0.00 | 0.00 | 0.00 | 0.00 | 0.00 | 0.00 |
| G2a1 | 0.00 | 0.00 | 0.00 | 0.00 | 0.00 | 7.41 | 0.00 | 0.00 | 0.00 | 2.94 | 0.00 |
| G2a1d2 | 0.00 | 0.00 | 0.00 | 1.85 | 0.00 | 0.00 | 0.00 | 0.00 | 0.00 | 0.00 | 0.00 |
| G2a1d2a | 0.97 | 0.00 | 0.00 | 0.00 | 0.00 | 0.00 | 0.00 | 0.00 | 0.00 | 0.00 | 0.00 |
| G2a1f | 0.97 | 0.00 | 0.00 | 0.00 | 0.00 | 0.00 | 0.00 | 0.00 | 0.00 | 0.00 | 0.00 |
| G2a2 | 0.00 | 1.47 | 0.00 | 3.70 | 0.00 | 0.00 | 0.00 | 0.00 | 0.00 | 0.00 | 0.00 |
| G2a3 | 0.00 | 0.00 | 0.00 | 0.00 | 0.00 | 3.70 | 0.00 | 0.00 | 0.00 | 0.00 | 0.00 |
| G2b1a2 | 0.97 | 0.00 | 0.00 | 0.00 | 0.00 | 0.00 | 0.00 | 0.00 | 0.00 | 0.00 | 0.00 |
| G2b1b | 0.00 | 0.00 | 0.00 | 0.00 | 0.00 | 0.00 | 0.00 | 0.00 | 0.00 | 1.47 | 0.00 |
| G2b2 | 0.00 | 1.47 | 0.00 | 0.00 | 0.00 | 0.00 | 0.00 | 0.00 | 0.00 | 0.00 | 0.00 |
| G3a1 | 0.00 | 0.00 | 1.25 | 0.00 | 0.00 | 0.00 | 0.00 | 1.16 | 0.00 | 1.47 | 0.00 |
| G3a1a | 0.00 | 0.00 | 0.00 | 0.00 | 0.00 | 0.00 | 0.00 | 0.00 | 1.32 | 1.47 | 0.00 |
| G3a3 | 0.00 | 0.00 | 0.00 | 0.00 | 0.00 | 3.70 | 0.00 | 0.00 | 0.00 | 0.00 | 0.00 |
| G3b | 0.00 | 0.00 | 0.00 | 0.00 | 0.00 | 0.00 | 0.00 | 0.00 | 0.00 | 1.47 | 0.00 |
| G3b1 | 0.00 | 0.00 | 0.00 | 0.00 | 0.00 | 0.00 | 0.00 | 0.00 | 0.00 | 1.47 | 0.00 |
| H | 0.00 | 0.00 | 0.00 | 1.85 | 7.14 | 0.00 | 2.00 | 2.33 | 0.00 | 0.00 | 9.09 |
| H10a1 | 0.00 | 0.00 | 0.00 | 0.00 | 0.00 | 0.00 | 0.00 | 0.00 | 1.32 | 0.00 | 0.00 |
| H13a2b2a | 0.00 | 0.00 | 0.00 | 1.85 | 0.00 | 0.00 | 0.00 | 0.00 | 0.00 | 0.00 | 0.00 |
| H14a | 0.00 | 0.00 | 5.00 | 0.00 | 0.00 | 0.00 | 0.00 | 0.00 | 0.00 | 0.00 | 7.58 |
| H15a1a1 | 0.00 | 0.00 | 1.25 | 0.00 | 0.00 | 0.00 | 10.00 | 0.00 | 0.00 | 0.00 | 0.00 |

| | | | | | | | | | | | |
|---|---|---|---|---|---|---|---|---|---|---|---|
| H15a1b | 0.00 | 0.00 | 1.25 | 0.00 | 0.00 | 0.00 | 8.00 | 3.49 | 0.00 | 0.00 | 0.00 |
| H16a1 | 0.00 | 1.47 | 0.00 | 0.00 | 0.00 | 0.00 | 2.00 | 0.00 | 0.00 | 0.00 | 0.00 |
| H1ae | 0.00 | 0.00 | 0.00 | 0.00 | 0.00 | 0.00 | 0.00 | 2.33 | 0.00 | 0.00 | 1.52 |
| H1af1 | 0.00 | 0.00 | 0.00 | 0.00 | 0.00 | 3.70 | 0.00 | 0.00 | 0.00 | 0.00 | 0.00 |
| H20 | 0.00 | 0.00 | 1.25 | 0.00 | 0.00 | 0.00 | 0.00 | 0.00 | 0.00 | 0.00 | 0.00 |
| H2a1 | 0.00 | 0.00 | 0.00 | 0.00 | 7.14 | 3.70 | 0.00 | 0.00 | 0.00 | 0.00 | 0.00 |
| H2a1a | 0.00 | 1.47 | 1.25 | 1.85 | 0.00 | 0.00 | 10.00 | 5.81 | 0.00 | 0.00 | 9.09 |
| H2a1f | 0.00 | 0.00 | 0.00 | 0.00 | 0.00 | 0.00 | 4.00 | 0.00 | 0.00 | 0.00 | 0.00 |
| H2b | 0.00 | 0.00 | 0.00 | 0.00 | 0.00 | 0.00 | 2.00 | 4.65 | 0.00 | 0.00 | 0.00 |
| H31b | 0.00 | 0.00 | 1.25 | 0.00 | 0.00 | 0.00 | 0.00 | 0.00 | 0.00 | 0.00 | 0.00 |
| H41a | 0.00 | 1.47 | 0.00 | 0.00 | 0.00 | 0.00 | 2.00 | 0.00 | 0.00 | 0.00 | 0.00 |
| H5a1 | 0.00 | 0.00 | 0.00 | 1.85 | 0.00 | 0.00 | 2.00 | 0.00 | 0.00 | 0.00 | 0.00 |
| H5a2a | 0.00 | 0.00 | 0.00 | 1.85 | 0.00 | 0.00 | 0.00 | 0.00 | 0.00 | 0.00 | 0.00 |
| H5b | 0.00 | 0.00 | 3.75 | 0.00 | 3.57 | 0.00 | 0.00 | 0.00 | 0.00 | 0.00 | 3.03 |
| H5e1 | 0.00 | 0.00 | 0.00 | 0.00 | 0.00 | 0.00 | 2.00 | 0.00 | 0.00 | 0.00 | 3.03 |
| H6a1a | 0.00 | 0.00 | 0.00 | 0.00 | 0.00 | 0.00 | 0.00 | 1.16 | 0.00 | 0.00 | 0.00 |
| H6a1b | 0.00 | 1.47 | 0.00 | 1.85 | 0.00 | 0.00 | 2.00 | 0.00 | 0.00 | 0.00 | 0.00 |
| H6b | 0.00 | 0.00 | 0.00 | 0.00 | 3.57 | 0.00 | 0.00 | 1.16 | 0.00 | 0.00 | 0.00 |
| H6b2 | 0.00 | 0.00 | 5.00 | 0.00 | 3.57 | 0.00 | 2.00 | 1.16 | 0.00 | 0.00 | 4.55 |
| H7b6 | 0.00 | 0.00 | 0.00 | 0.00 | 7.14 | 3.70 | 0.00 | 0.00 | 0.00 | 0.00 | 0.00 |
| H8b1 | 0.00 | 2.94 | 0.00 | 0.00 | 0.00 | 0.00 | 0.00 | 0.00 | 0.00 | 0.00 | 0.00 |
| HV13b | 0.00 | 0.00 | 0.00 | 0.00 | 0.00 | 0.00 | 0.00 | 1.16 | 0.00 | 0.00 | 0.00 |
| HV14a | 0.00 | 0.00 | 0.00 | 0.00 | 10.71 | 3.70 | 0.00 | 0.00 | 0.00 | 0.00 | 0.00 |
| HV17 | 0.00 | 1.47 | 0.00 | 0.00 | 0.00 | 0.00 | 0.00 | 0.00 | 0.00 | 0.00 | 0.00 |
| HV18 | 0.00 | 0.00 | 0.00 | 0.00 | 0.00 | 0.00 | 2.00 | 0.00 | 0.00 | 0.00 | 0.00 |
| HV1a3 | 0.00 | 0.00 | 0.00 | 0.00 | 3.57 | 0.00 | 0.00 | 0.00 | 0.00 | 0.00 | 0.00 |
| HV2 | 0.00 | 0.00 | 0.00 | 0.00 | 3.57 | 3.70 | 0.00 | 0.00 | 0.00 | 0.00 | 0.00 |
| HV2a1 | 0.00 | 0.00 | 0.00 | 0.00 | 0.00 | 0.00 | 0.00 | 1.16 | 0.00 | 0.00 | 0.00 |
| HV6 | 0.00 | 1.47 | 0.00 | 0.00 | 0.00 | 0.00 | 0.00 | 0.00 | 0.00 | 0.00 | 0.00 |
| HV8 | 0.00 | 0.00 | 0.00 | 0.00 | 0.00 | 0.00 | 2.00 | 0.00 | 0.00 | 0.00 | 0.00 |
| HV9 | 0.00 | 4.41 | 0.00 | 0.00 | 0.00 | 0.00 | 0.00 | 0.00 | 0.00 | 0.00 | 0.00 |
| I1b | 0.00 | 0.00 | 0.00 | 0.00 | 0.00 | 0.00 | 0.00 | 2.33 | 0.00 | 0.00 | 0.00 |
| I4b | 0.00 | 2.94 | 0.00 | 0.00 | 0.00 | 0.00 | 0.00 | 0.00 | 0.00 | 0.00 | 0.00 |
| J1b06 | 0.00 | 0.00 | 1.25 | 0.00 | 0.00 | 0.00 | 0.00 | 0.00 | 0.00 | 0.00 | 0.00 |

| | | | | | | | | | | | |
|---|---|---|---|---|---|---|---|---|---|---|---|
| J1b1 | 0.00 | 0.00 | 0.00 | 3.70 | 0.00 | 0.00 | 0.00 | 0.00 | 0.00 | 0.00 | 0.00 |
| J1b1a | 0.00 | 0.00 | 1.25 | 0.00 | 0.00 | 0.00 | 0.00 | 0.00 | 0.00 | 0.00 | 0.00 |
| J1b1a1 | 0.00 | 1.47 | 5.00 | 0.00 | 0.00 | 0.00 | 0.00 | 2.33 | 0.00 | 0.00 | 1.52 |
| J1b1b1 | 0.00 | 0.00 | 0.00 | 3.70 | 7.14 | 0.00 | 0.00 | 1.16 | 0.00 | 0.00 | 3.03 |
| J1b2 | 0.00 | 0.00 | 0.00 | 5.56 | 0.00 | 0.00 | 0.00 | 0.00 | 0.00 | 0.00 | 0.00 |
| J1b6 | 0.00 | 0.00 | 0.00 | 0.00 | 0.00 | 0.00 | 2.00 | 0.00 | 0.00 | 0.00 | 0.00 |
| J1c1b1a | 0.00 | 2.94 | 0.00 | 0.00 | 0.00 | 0.00 | 0.00 | 0.00 | 0.00 | 0.00 | 0.00 |
| J1d | 0.00 | 0.00 | 0.00 | 0.00 | 0.00 | 0.00 | 0.00 | 1.16 | 0.00 | 0.00 | 0.00 |
| J1d1 | 0.00 | 1.47 | 1.25 | 0.00 | 0.00 | 0.00 | 0.00 | 0.00 | 0.00 | 0.00 | 3.03 |
| J1d2 | 0.00 | 0.00 | 0.00 | 0.00 | 0.00 | 3.70 | 0.00 | 0.00 | 0.00 | 0.00 | 0.00 |
| J1d6a | 0.00 | 0.00 | 0.00 | 0.00 | 3.57 | 0.00 | 0.00 | 0.00 | 0.00 | 0.00 | 0.00 |
| J2b1a2a | 0.00 | 0.00 | 1.25 | 0.00 | 0.00 | 0.00 | 2.00 | 2.33 | 0.00 | 0.00 | 0.00 |
| K1a12 | 0.00 | 0.00 | 2.50 | 0.00 | 0.00 | 0.00 | 0.00 | 0.00 | 0.00 | 0.00 | 0.00 |
| K1a12a | 0.00 | 2.94 | 1.25 | 0.00 | 0.00 | 0.00 | 2.00 | 3.49 | 0.00 | 0.00 | 4.55 |
| K1a2a | 0.00 | 0.00 | 1.25 | 0.00 | 0.00 | 0.00 | 0.00 | 0.00 | 0.00 | 0.00 | 0.00 |
| K1a4i | 0.00 | 7.35 | 0.00 | 0.00 | 0.00 | 0.00 | 0.00 | 0.00 | 0.00 | 0.00 | 0.00 |
| K1b1a1 | 0.00 | 0.00 | 0.00 | 0.00 | 0.00 | 0.00 | 0.00 | 0.00 | 0.00 | 0.00 | 1.52 |
| K1b1a1a | 0.00 | 0.00 | 0.00 | 0.00 | 0.00 | 3.70 | 0.00 | 0.00 | 0.00 | 0.00 | 0.00 |
| K1b2a | 0.00 | 0.00 | 3.75 | 0.00 | 0.00 | 0.00 | 0.00 | 2.33 | 0.00 | 0.00 | 1.52 |
| K3 | 0.97 | 0.00 | 0.00 | 0.00 | 0.00 | 0.00 | 0.00 | 0.00 | 0.00 | 0.00 | 0.00 |
| M10a1a | 0.97 | 0.00 | 0.00 | 0.00 | 0.00 | 0.00 | 0.00 | 0.00 | 0.00 | 0.00 | 0.00 |
| M10a1a1 | 0.97 | 0.00 | 0.00 | 0.00 | 0.00 | 0.00 | 0.00 | 0.00 | 0.00 | 0.00 | 0.00 |
| M10a1b | 0.97 | 0.00 | 0.00 | 0.00 | 0.00 | 0.00 | 0.00 | 0.00 | 0.00 | 0.00 | 0.00 |
| M11a2 | 0.00 | 0.00 | 0.00 | 0.00 | 0.00 | 0.00 | 0.00 | 0.00 | 1.32 | 1.47 | 0.00 |
| M11b | 0.00 | 0.00 | 0.00 | 0.00 | 3.57 | 0.00 | 0.00 | 0.00 | 0.00 | 0.00 | 0.00 |
| M11c | 0.97 | 0.00 | 0.00 | 0.00 | 0.00 | 0.00 | 0.00 | 0.00 | 0.00 | 0.00 | 0.00 |
| M13a1b | 0.00 | 0.00 | 0.00 | 0.00 | 0.00 | 0.00 | 0.00 | 0.00 | 0.00 | 1.47 | 0.00 |
| M13a2 | 0.00 | 0.00 | 0.00 | 0.00 | 0.00 | 0.00 | 0.00 | 0.00 | 2.63 | 5.88 | 0.00 |
| M13b2 | 0.00 | 0.00 | 0.00 | 0.00 | 0.00 | 0.00 | 0.00 | 0.00 | 0.00 | 1.47 | 0.00 |
| M30 | 0.00 | 2.94 | 0.00 | 0.00 | 0.00 | 0.00 | 0.00 | 1.16 | 0.00 | 0.00 | 1.52 |
| M30a | 0.00 | 0.00 | 0.00 | 0.00 | 0.00 | 0.00 | 0.00 | 0.00 | 0.00 | 1.47 | 0.00 |
| M30c1 | 0.00 | 0.00 | 0.00 | 0.00 | 3.57 | 0.00 | 0.00 | 0.00 | 0.00 | 0.00 | 0.00 |
| M33a | 0.00 | 0.00 | 0.00 | 0.00 | 7.14 | 0.00 | 0.00 | 0.00 | 0.00 | 0.00 | 0.00 |
| M35a | 0.00 | 0.00 | 0.00 | 0.00 | 0.00 | 0.00 | 0.00 | 1.16 | 0.00 | 0.00 | 0.00 |

| | | | | | | | | | | | |
|---|---|---|---|---|---|---|---|---|---|---|---|
| M38 | 0.00 | 0.00 | 0.00 | 0.00 | 0.00 | 0.00 | 0.00 | 0.00 | 1.32 | 0.00 | 0.00 |
| M3a1b | 0.00 | 0.00 | 3.75 | 0.00 | 0.00 | 0.00 | 0.00 | 2.33 | 0.00 | 0.00 | 1.52 |
| M3d1a1 | 0.00 | 0.00 | 0.00 | 0.00 | 0.00 | 0.00 | 0.00 | 0.00 | 3.95 | 0.00 | 0.00 |
| M49a1 | 0.00 | 0.00 | 0.00 | 0.00 | 0.00 | 0.00 | 0.00 | 0.00 | 0.00 | 1.47 | 0.00 |
| M54 | 0.00 | 0.00 | 0.00 | 0.00 | 0.00 | 0.00 | 0.00 | 0.00 | 2.63 | 0.00 | 0.00 |
| M5a | 0.00 | 1.47 | 0.00 | 0.00 | 0.00 | 0.00 | 0.00 | 0.00 | 0.00 | 0.00 | 0.00 |
| M5b2b1a | 0.00 | 0.00 | 0.00 | 0.00 | 0.00 | 0.00 | 0.00 | 0.00 | 1.32 | 0.00 | 0.00 |
| M5c2 | 0.00 | 0.00 | 0.00 | 0.00 | 0.00 | 0.00 | 0.00 | 0.00 | 1.32 | 0.00 | 0.00 |
| M61a | 0.00 | 0.00 | 0.00 | 0.00 | 0.00 | 0.00 | 0.00 | 0.00 | 0.00 | 1.47 | 0.00 |
| M62b | 0.00 | 0.00 | 0.00 | 0.00 | 0.00 | 0.00 | 0.00 | 0.00 | 0.00 | 2.94 | 0.00 |
| M62b1a | 0.00 | 0.00 | 0.00 | 0.00 | 0.00 | 0.00 | 0.00 | 0.00 | 0.00 | 1.47 | 0.00 |
| M62b1a1 | 0.00 | 0.00 | 0.00 | 0.00 | 0.00 | 0.00 | 0.00 | 0.00 | 0.00 | 7.35 | 0.00 |
| M62b2 | 0.00 | 0.00 | 0.00 | 0.00 | 0.00 | 0.00 | 0.00 | 0.00 | 0.00 | 5.88 | 0.00 |
| M65a | 0.00 | 0.00 | 0.00 | 0.00 | 0.00 | 0.00 | 2.00 | 2.33 | 0.00 | 0.00 | 0.00 |
| M65a2 | 0.00 | 0.00 | 0.00 | 0.00 | 0.00 | 0.00 | 0.00 | 0.00 | 0.00 | 1.47 | 0.00 |
| M70 | 0.00 | 0.00 | 0.00 | 0.00 | 0.00 | 0.00 | 0.00 | 0.00 | 1.32 | 2.94 | 0.00 |
| M7b1a1 | 0.97 | 0.00 | 0.00 | 0.00 | 0.00 | 0.00 | 0.00 | 0.00 | 0.00 | 0.00 | 0.00 |
| M7b1a1a | 0.97 | 0.00 | 0.00 | 0.00 | 0.00 | 0.00 | 0.00 | 0.00 | 0.00 | 0.00 | 0.00 |
| M7b1a1a1 | 0.97 | 0.00 | 0.00 | 0.00 | 0.00 | 0.00 | 0.00 | 0.00 | 0.00 | 0.00 | 0.00 |
| M7b1a1b | 0.00 | 0.00 | 0.00 | 1.85 | 0.00 | 0.00 | 0.00 | 0.00 | 0.00 | 0.00 | 0.00 |
| M7b1a1c | 0.97 | 0.00 | 0.00 | 0.00 | 0.00 | 3.70 | 0.00 | 0.00 | 0.00 | 0.00 | 0.00 |
| M7b1a1g | 0.97 | 0.00 | 0.00 | 0.00 | 0.00 | 0.00 | 0.00 | 0.00 | 0.00 | 0.00 | 0.00 |
| M7b1a2a | 0.00 | 2.94 | 0.00 | 0.00 | 0.00 | 0.00 | 0.00 | 0.00 | 0.00 | 0.00 | 0.00 |
| M7c1 | 0.00 | 0.00 | 1.25 | 0.00 | 0.00 | 0.00 | 0.00 | 1.16 | 0.00 | 0.00 | 0.00 |
| M7c1a3 | 0.97 | 0.00 | 0.00 | 0.00 | 0.00 | 0.00 | 0.00 | 0.00 | 0.00 | 0.00 | 0.00 |
| M7c1a3a | 0.97 | 0.00 | 0.00 | 0.00 | 0.00 | 0.00 | 0.00 | 0.00 | 0.00 | 0.00 | 0.00 |
| M7c1b2a | 0.97 | 0.00 | 0.00 | 0.00 | 0.00 | 0.00 | 0.00 | 0.00 | 0.00 | 0.00 | 0.00 |
| M7c1b2b | 0.97 | 0.00 | 0.00 | 0.00 | 0.00 | 0.00 | 0.00 | 0.00 | 0.00 | 0.00 | 0.00 |
| M8a2 | 0.97 | 0.00 | 0.00 | 0.00 | 0.00 | 0.00 | 0.00 | 0.00 | 0.00 | 0.00 | 0.00 |
| M8a2a1 | 0.97 | 0.00 | 0.00 | 0.00 | 0.00 | 0.00 | 0.00 | 0.00 | 0.00 | 0.00 | 0.00 |
| M8a3 | 0.97 | 0.00 | 0.00 | 1.85 | 0.00 | 0.00 | 0.00 | 0.00 | 0.00 | 0.00 | 0.00 |
| M8a3a | 1.94 | 0.00 | 0.00 | 0.00 | 0.00 | 0.00 | 0.00 | 0.00 | 0.00 | 0.00 | 0.00 |
| M91a | 0.00 | 0.00 | 0.00 | 0.00 | 0.00 | 0.00 | 0.00 | 0.00 | 0.00 | 1.47 | 0.00 |
| M9a1a1a | 0.97 | 0.00 | 0.00 | 0.00 | 0.00 | 0.00 | 0.00 | 0.00 | 0.00 | 0.00 | 0.00 |

| | | | | | | | | | | | |
|---|---|---|---|---|---|---|---|---|---|---|---|
| M9a1a1c1b1a | 0.00 | 0.00 | 0.00 | 0.00 | 0.00 | 0.00 | 0.00 | 0.00 | 13.16 | 2.94 | 0.00 |
| M9a1a1c1b1a1 | 0.00 | 0.00 | 0.00 | 0.00 | 0.00 | 0.00 | 0.00 | 0.00 | 1.32 | 0.00 | 0.00 |
| M9a1a2 | 0.00 | 0.00 | 0.00 | 0.00 | 0.00 | 0.00 | 0.00 | 0.00 | 3.95 | 7.35 | 0.00 |
| M9a1b1 | 0.00 | 0.00 | 0.00 | 1.85 | 0.00 | 0.00 | 0.00 | 0.00 | 0.00 | 2.94 | 0.00 |
| M9a1b1c | 0.00 | 0.00 | 0.00 | 0.00 | 0.00 | 0.00 | 0.00 | 0.00 | 0.00 | 2.94 | 0.00 |
| N10a | 0.97 | 0.00 | 0.00 | 0.00 | 0.00 | 0.00 | 0.00 | 0.00 | 0.00 | 0.00 | 0.00 |
| N11a1 | 0.00 | 0.00 | 0.00 | 0.00 | 0.00 | 0.00 | 0.00 | 0.00 | 0.00 | 1.47 | 0.00 |
| N1a1b1 | 0.00 | 0.00 | 1.25 | 0.00 | 0.00 | 0.00 | 0.00 | 2.33 | 0.00 | 0.00 | 0.00 |
| N9a | 0.97 | 0.00 | 0.00 | 0.00 | 0.00 | 0.00 | 0.00 | 0.00 | 0.00 | 0.00 | 0.00 |
| N9a1 | 0.00 | 2.94 | 0.00 | 0.00 | 0.00 | 0.00 | 0.00 | 0.00 | 0.00 | 0.00 | 0.00 |
| N9a2a | 0.97 | 0.00 | 0.00 | 0.00 | 0.00 | 0.00 | 0.00 | 0.00 | 0.00 | 0.00 | 0.00 |
| N9a3 | 0.97 | 4.41 | 0.00 | 0.00 | 0.00 | 3.70 | 0.00 | 0.00 | 0.00 | 0.00 | 0.00 |
| N9a4b1 | 1.94 | 0.00 | 0.00 | 0.00 | 0.00 | 0.00 | 0.00 | 0.00 | 0.00 | 0.00 | 0.00 |
| N9a9 | 1.94 | 0.00 | 0.00 | 0.00 | 0.00 | 0.00 | 0.00 | 0.00 | 0.00 | 0.00 | 0.00 |
| R0a | 0.00 | 0.00 | 2.50 | 0.00 | 0.00 | 0.00 | 0.00 | 0.00 | 0.00 | 0.00 | 3.03 |
| R0a`b | 0.00 | 0.00 | 1.25 | 0.00 | 0.00 | 0.00 | 0.00 | 0.00 | 0.00 | 0.00 | 0.00 |
| R11b1a | 0.97 | 0.00 | 0.00 | 0.00 | 0.00 | 0.00 | 0.00 | 0.00 | 0.00 | 0.00 | 0.00 |
| R11b1b | 0.97 | 0.00 | 0.00 | 0.00 | 0.00 | 0.00 | 0.00 | 0.00 | 0.00 | 0.00 | 0.00 |
| R1a1 | 0.00 | 0.00 | 0.00 | 1.85 | 0.00 | 0.00 | 0.00 | 0.00 | 0.00 | 0.00 | 0.00 |
| R2 | 0.00 | 0.00 | 3.75 | 0.00 | 3.57 | 3.70 | 0.00 | 0.00 | 0.00 | 0.00 | 6.06 |
| R2d | 0.00 | 0.00 | 1.25 | 0.00 | 0.00 | 0.00 | 0.00 | 0.00 | 0.00 | 0.00 | 0.00 |
| T | 0.00 | 0.00 | 1.25 | 0.00 | 0.00 | 0.00 | 0.00 | 0.00 | 0.00 | 0.00 | 0.00 |
| T1a | 0.00 | 0.00 | 0.00 | 0.00 | 0.00 | 0.00 | 0.00 | 0.00 | 0.00 | 0.00 | 1.52 |
| T1a1b | 0.00 | 0.00 | 0.00 | 1.85 | 0.00 | 0.00 | 0.00 | 0.00 | 0.00 | 0.00 | 0.00 |
| T1a1b1 | 0.00 | 2.94 | 0.00 | 0.00 | 0.00 | 0.00 | 0.00 | 0.00 | 0.00 | 0.00 | 3.03 |
| T1a1n | 0.00 | 0.00 | 3.75 | 0.00 | 0.00 | 0.00 | 6.00 | 1.16 | 0.00 | 0.00 | 3.03 |
| T2 | 0.00 | 0.00 | 1.25 | 0.00 | 3.57 | 0.00 | 2.00 | 0.00 | 0.00 | 0.00 | 0.00 |
| T2a1a | 0.00 | 0.00 | 0.00 | 0.00 | 0.00 | 0.00 | 4.00 | 0.00 | 0.00 | 0.00 | 0.00 |
| T2a1b | 0.00 | 0.00 | 0.00 | 0.00 | 3.57 | 0.00 | 0.00 | 0.00 | 0.00 | 0.00 | 0.00 |
| T2b34 | 0.00 | 0.00 | 0.00 | 0.00 | 0.00 | 0.00 | 0.00 | 1.16 | 0.00 | 0.00 | 0.00 |
| T2d1b | 0.00 | 0.00 | 5.00 | 0.00 | 0.00 | 0.00 | 0.00 | 0.00 | 0.00 | 0.00 | 1.52 |
| T2d2 | 0.00 | 0.00 | 0.00 | 0.00 | 0.00 | 0.00 | 2.00 | 0.00 | 0.00 | 0.00 | 0.00 |
| T2g | 0.00 | 2.94 | 0.00 | 0.00 | 0.00 | 0.00 | 0.00 | 0.00 | 0.00 | 0.00 | 0.00 |
| T2g1b | 0.00 | 0.00 | 0.00 | 0.00 | 3.57 | 0.00 | 0.00 | 0.00 | 0.00 | 0.00 | 0.00 |

| | | | | | | | | | | | |
|---|---|---|---|---|---|---|---|---|---|---|---|
| U1a1 | 0.00 | 0.00 | 0.00 | 1.85 | 0.00 | 0.00 | 0.00 | 0.00 | 0.00 | 0.00 | 0.00 |
| U1a1c1a | 0.00 | 0.00 | 0.00 | 0.00 | 0.00 | 0.00 | 0.00 | 0.00 | 0.00 | 1.47 | 0.00 |
| U2a2 | 0.00 | 0.00 | 0.00 | 0.00 | 0.00 | 0.00 | 0.00 | 0.00 | 1.32 | 0.00 | 0.00 |
| U2b | 0.00 | 1.47 | 0.00 | 0.00 | 0.00 | 0.00 | 0.00 | 0.00 | 0.00 | 0.00 | 0.00 |
| U2b1a | 0.00 | 0.00 | 0.00 | 0.00 | 0.00 | 0.00 | 0.00 | 0.00 | 3.95 | 0.00 | 0.00 |
| U2b2 | 0.00 | 0.00 | 3.75 | 0.00 | 0.00 | 0.00 | 0.00 | 5.81 | 0.00 | 0.00 | 1.52 |
| U2c1b | 0.00 | 0.00 | 0.00 | 0.00 | 0.00 | 3.70 | 0.00 | 0.00 | 0.00 | 0.00 | 0.00 |
| U2e1 | 0.00 | 0.00 | 0.00 | 0.00 | 0.00 | 0.00 | 2.00 | 0.00 | 0.00 | 0.00 | 0.00 |
| U2e1h | 0.00 | 0.00 | 0.00 | 1.85 | 0.00 | 0.00 | 8.00 | 2.33 | 0.00 | 0.00 | 1.52 |
| U2e3a | 0.00 | 0.00 | 0.00 | 0.00 | 0.00 | 0.00 | 0.00 | 1.16 | 0.00 | 0.00 | 0.00 |
| U3 | 0.00 | 0.00 | 0.00 | 0.00 | 0.00 | 0.00 | 0.00 | 1.16 | 0.00 | 0.00 | 0.00 |
| U4a1 | 0.00 | 1.47 | 0.00 | 0.00 | 0.00 | 0.00 | 0.00 | 2.33 | 0.00 | 0.00 | 0.00 |
| U4a2 | 0.00 | 0.00 | 0.00 | 0.00 | 0.00 | 3.70 | 0.00 | 0.00 | 0.00 | 0.00 | 0.00 |
| U4b | 0.00 | 0.00 | 0.00 | 5.56 | 0.00 | 0.00 | 0.00 | 0.00 | 0.00 | 0.00 | 0.00 |
| U4b1a1a1 | 0.00 | 0.00 | 2.50 | 0.00 | 0.00 | 0.00 | 0.00 | 1.16 | 0.00 | 0.00 | 0.00 |
| U4b1a4 | 0.00 | 0.00 | 0.00 | 0.00 | 3.57 | 0.00 | 0.00 | 0.00 | 0.00 | 0.00 | 0.00 |
| U4b2 | 0.00 | 0.00 | 10.00 | 0.00 | 0.00 | 0.00 | 0.00 | 3.49 | 0.00 | 0.00 | 3.03 |
| U4c1 | 0.00 | 0.00 | 0.00 | 0.00 | 3.57 | 0.00 | 0.00 | 0.00 | 0.00 | 0.00 | 0.00 |
| U5a1a1 | 0.00 | 0.00 | 1.25 | 0.00 | 0.00 | 0.00 | 0.00 | 1.16 | 0.00 | 0.00 | 0.00 |
| U5a1b | 0.00 | 1.47 | 0.00 | 0.00 | 0.00 | 0.00 | 0.00 | 1.16 | 0.00 | 0.00 | 0.00 |
| U5a1b1 | 0.00 | 0.00 | 0.00 | 1.85 | 0.00 | 0.00 | 0.00 | 0.00 | 0.00 | 0.00 | 0.00 |
| U5a1d2b | 0.00 | 0.00 | 0.00 | 0.00 | 0.00 | 0.00 | 0.00 | 1.16 | 0.00 | 0.00 | 0.00 |
| U5a1g | 0.00 | 1.47 | 0.00 | 0.00 | 0.00 | 0.00 | 0.00 | 0.00 | 0.00 | 0.00 | 0.00 |
| U5a2a1 | 0.00 | 1.47 | 1.25 | 1.85 | 0.00 | 0.00 | 2.00 | 1.16 | 0.00 | 0.00 | 0.00 |
| U5a2b | 0.00 | 0.00 | 2.50 | 0.00 | 0.00 | 0.00 | 0.00 | 1.16 | 0.00 | 0.00 | 1.52 |
| U5b2 | 0.00 | 1.47 | 0.00 | 0.00 | 0.00 | 0.00 | 0.00 | 0.00 | 0.00 | 0.00 | 0.00 |
| U5b2a1b | 0.00 | 0.00 | 0.00 | 0.00 | 0.00 | 0.00 | 0.00 | 1.16 | 0.00 | 0.00 | 0.00 |
| U7a3a | 0.00 | 0.00 | 0.00 | 0.00 | 0.00 | 0.00 | 0.00 | 1.16 | 0.00 | 0.00 | 3.03 |
| U7a3b | 0.00 | 0.00 | 0.00 | 0.00 | 0.00 | 0.00 | 0.00 | 1.16 | 0.00 | 0.00 | 0.00 |
| V1a1 | 0.00 | 0.00 | 0.00 | 1.85 | 0.00 | 0.00 | 0.00 | 0.00 | 0.00 | 0.00 | 0.00 |
| W | 0.00 | 0.00 | 0.00 | 0.00 | 0.00 | 0.00 | 0.00 | 0.00 | 1.32 | 0.00 | 0.00 |
| W1c | 0.00 | 0.00 | 1.25 | 0.00 | 0.00 | 0.00 | 0.00 | 0.00 | 0.00 | 0.00 | 0.00 |
| W3 | 0.00 | 0.00 | 1.25 | 0.00 | 0.00 | 0.00 | 0.00 | 0.00 | 0.00 | 0.00 | 0.00 |
| W3b | 0.00 | 0.00 | 2.50 | 0.00 | 0.00 | 0.00 | 4.00 | 0.00 | 0.00 | 0.00 | 12.12 |

| | | | | | | | | | | | |
|---|---|---|---|---|---|---|---|---|---|---|---|
| W6 | 0.00 | 0.00 | 0.00 | 0.00 | 3.57 | 0.00 | 0.00 | 0.00 | 0.00 | 0.00 | 0.00 |
| X2 | 0.00 | 0.00 | 2.50 | 0.00 | 0.00 | 0.00 | 0.00 | 1.16 | 0.00 | 0.00 | 0.00 |
| X2c | 0.00 | 0.00 | 2.50 | 0.00 | 0.00 | 0.00 | 0.00 | 0.00 | 0.00 | 0.00 | 0.00 |
| X2e2a | 0.00 | 0.00 | 0.00 | 0.00 | 0.00 | 0.00 | 0.00 | 1.16 | 0.00 | 0.00 | 0.00 |
| X2i | 0.00 | 0.00 | 0.00 | 1.85 | 0.00 | 0.00 | 0.00 | 0.00 | 0.00 | 0.00 | 0.00 |
| X2p | 0.00 | 0.00 | 0.00 | 0.00 | 0.00 | 0.00 | 0.00 | 1.16 | 0.00 | 0.00 | 0.00 |
| Z3c | 0.00 | 1.47 | 0.00 | 0.00 | 0.00 | 0.00 | 0.00 | 0.00 | 0.00 | 0.00 | 0.00 |
| Z3d | 0.97 | 0.00 | 0.00 | 0.00 | 0.00 | 0.00 | 0.00 | 0.00 | 0.00 | 0.00 | 0.00 |
| Z7 | 0.00 | 0.00 | 0.00 | 0.00 | 0.00 | 0.00 | 0.00 | 0.00 | 0.00 | 1.47 | 0.00 |
