# Supplementary material for: Mitochondrial DNA genomes revealed different patterns of high-altitude adaptation in high-altitude Tajiks compared with Tibetans and Sherpas
Source: Sci Rep. 2020 Jun 29;10:10592. doi: 10.1038/s41598-020-67519-z (PMC7324373; doi:10.1038/s41598-020-67519-z)
Supplement: Supplementary file 3 — Supplementary Table 3. [file 41598_2020_67519_MOESM3_ESM.pdf]

Mitochondrial DNA genomes revealed different patterns of high-altitude adaptation in high-altitude Tajiks compared with Tibetans and Sherpas

Yu Chen[1], Liang Gong[1], Xinyuan Liu[1], Xingshu Chen[1], Shenghong Yang[2#], Yongjun Luo[1#]

(1 Department of Military Medical Geography, Army Health Service Training Base, Third Military Medical University(Army Medical University), Chongqing 40038, China; 2 Health Department of the 957th Hospital of PLA, Ali, Tibet, China 859000)

[#]Corresponding author:

Prof. Shenghong Yang, Health Department of the 957th Hospital of PLA, Ali, Tibet, 859000, China.

(E-mail: kevinysh0751@163.com)

Prof. Yongjun Luo, Army Health Service Training Base, Third Military Medical University(Army Medical University), Chongqing 400038, China.

(E-mail: luo.yongjun@qq.com)

Table S3 Results of p values between 0.025 and 0.0000001 in comparison of mtDNA genome between Tibetan and HA-Tajik

Alleles	Amino acid change	Regions	Tibetan		HA-Tajik		χ^2	p values
			Variants	non-variants	Variants	non-variants		
3594T	No	ND1	16	52	40	40	10.95	0.001
4104G	No	ND1	16	52	40	40	10.95	0.001
4216C	Yes	ND1	0	68	20	60	19.66	0.000009
4561C	Yes	ND2	12	56	0	80	15.36	0.000089
4646C	No	ND2	0	68	10	70	7.24	0.007
4769G	Yes	ND2	52	16	42	38	9.11	0.003
7028T	No	COX1	52	16	43	37	8.26	0.004
7146G	Yes	COX1	16	52	40	40	10.95	0.001
7256T	No	COX1	17	51	40	40	9.7	0.002
7664A	Yes	COX2	12	56	0	80	15.36	0.000089
8149G	No	COX2	12	56	0	80	15.36	0.000089
8468T	No	ATP8	16	52	40	40	10.95	0.001
8655T	No	ATP6	16	52	40	40	10.95	0.001
8860G	Yes	ATP6	52	16	40	40	10.95	0.001
10664T	No	ND4L	16	52	40	40	10.95	0.001
10688A	No	ND4L	16	52	40	40	10.95	0.001
10810C	No	ND4	16	52	40	40	10.95	0.001
10915C	No	ND4	16	52	40	40	10.95	0.001
11251G	No	ND4	0	68	17	63	16.33	0.000053
11467G	No	ND4	1	67	23	57	20.13	0.000007
11914A	No	ND4	18	50	41	39	9.42	0.002
12372A	No	ND5	1	67	24	56	21.31	0.000004
13105G	Yes	ND5	16	52	40	40	10.95	0.001
13276G	Yes	ND5	16	52	40	40	10.95	0.001
13506T	No	ND5	16	52	40	40	10.95	0.001
13650T	No	ND5	16	52	40	40	10.95	0.001
14766T	Yes	CYTB	52	16	43	37	8.26	0.004
15326G	Yes	CYTB	51	17	40	40	9.7	0.002
15452A	Yes	CYTB	0	68	16	64	15.25	0.000094
15510G	Yes	CYTB	12	56	0	80	15.36	0.000089
15607G	No	CYTB	0	68	9	71	6.3	0.012
15721C	No	CYTB	12	56	0	80	15.36	0.000089
