# Supplementary material for: Mitochondrial DNA genomes revealed different patterns of high-altitude adaptation in high-altitude Tajiks compared with Tibetans and Sherpas
Source: Sci Rep. 2020 Jun 29;10:10592. doi: 10.1038/s41598-020-67519-z (PMC7324373; doi:10.1038/s41598-020-67519-z)
Supplement: Supplementary file 4 — Supplementary Table 4. [file 41598_2020_67519_MOESM4_ESM.pdf]

Mitochondrial DNA genomes revealed different patterns of high-altitude adaptation in high-altitude Tajiks compared with Tibetans and Sherpas

Yu Chen[1], Liang Gong[1], Xinyuan Liu[1], Xingshu Chen[1], Shenghong Yang[2#], Yongjun Luo[1#]

(1 Department of Military Medical Geography, Army Health Service Training Base, Third Military Medical University(Army Medical University), Chongqing 40038, China; 2 Health Department of the 957th Hospital of PLA, Ali, Tibet, China 859000)

[#]Corresponding author:

Prof. Shenghong Yang, Health Department of the 957th Hospital of PLA, Ali, Tibet, 859000, China.

(E-mail: kevinysh0751@163.com)

Prof. Yongjun Luo, Army Health Service Training Base, Third Military Medical University(Army Medical University), Chongqing 400038, China.

(E-mail: luo.yongjun@qq.com)

Table S4 Results of p values between 0.025 and 0.0000001 in comparison of mtDNA genome between Sherpa and HA-Tajik

Alleles	Amino acid change	Regions	Sherpa		HA-Tajik		χ^2	p values
			variants	non-variants	variants	non-variants		
3394C	No	ND1	14	62	0	80	16.19	0.000057
3552A	No	ND1	14	62	1	79	13.22	0.000277
3745A	Yes	ND1	11	65	2	78	7.32	0.007
4248C	No	ND1	20	56	4	76	13.6	0.000226
4491A	Yes	ND2	14	62	0	80	16.19	0.000057
4646C	No	ND2	0	76	10	70	8.17	0.004
4696C	Yes	ND2	14	62	0	80	16.19	0.000057
4715G	No	ND2	14	62	1	79	13.22	0.000277
4824G	Yes	ND2	20	56	0	80	24.15	0.000001
4883T	No	ND2	8	68	0	80	6.85	0.009
4917G	Yes	ND2	0	76	9	71	7.12	0.008
5178A	Yes	ND2	8	68	0	80	6.85	0.009
5460A	Yes	ND2	1	75	12	68	9.55	0.002
5999C	No	COX1	0	76	10	70	8.17	0.004
6026A	No	COX1	14	62	1	79	13.22	0.000277
6047G	No	COX1	0	76	10	70	8.17	0.004
7142C	No	COX1	11	65	0	80	12.46	0.000416
7196A	No	COX1	14	62	1	79	13.22	0.000277
7697A	Yes	COX2	11	65	0	80	12.46	0.000416
7705C	No	COX2	0	76	10	70	8.17	0.004
8459G	No	ATP8	14	62	0	80	16.19	0.000057
8584A	Yes	ATP6	14	62	1	79	13.22	0.000277
8794T	Yes	ATP6	20	56	0	80	24.15	0.000001
9052G	Yes	ATP6	14	62	0	80	16.19	0.000057
9242G	No	COX2	11	65	0	80	12.46	0.000416
9545G	No	COX2	16	60	1	79	15.74	0.000073
10304C	No	ND3	14	62	0	80	16.19	0.000057
11467G	No	ND4	4	72	23	57	15.02	0.000106
11914A	No	ND4	15	61	41	39	16.82	0.000041
11969A	Yes	ND4	15	61	1	79	14.47	0.000142
12372A	No	ND5	4	72	24	56	16.19	0.000057
12624C	No	ND5	14	62	0	80	16.19	0.000057
12672G	No	ND5	14	62	1	79	13.22	0.000277
13111C	No	ND5	14	62	0	80	16.19	0.000057
13263G	No	ND5	14	62	1	79	13.22	0.000277
13563G	No	ND5	11	65	0	80	12.46	0.000416
13708A	Yes	ND5	3	73	14	66	7.37	0.007
14308C	No	ND6	14	62	0	80	16.19	0.000057
14318C	Yes	ND6	14	62	1	79	13.22	0.000277

14417G	Yes	ND6	10	66	0	80	9.16	0.002
15204C	Yes	CYTB	14	62	1	79	13.22	0.000277
15452A	Yes	CYTB	0	76	16	64	16.94	0.000039
15487T	No	CYTB	15	61	1	79	14.47	0.000142
