# Supplementary material for: Mitochondrial DNA genomes revealed different patterns of high-altitude adaptation in high-altitude Tajiks compared with Tibetans and Sherpas
Source: Sci Rep. 2020 Jun 29;10:10592. doi: 10.1038/s41598-020-67519-z (PMC7324373; doi:10.1038/s41598-020-67519-z)
Supplement: Supplementary file 5 — Supplementary Table 5. [file 41598_2020_67519_MOESM5_ESM.pdf]

Mitochondrial DNA genomes revealed different patterns of high-altitude adaptation in high-altitude Tajiks compared with Tibetans and Sherpas

Yu Chen[1], Liang Gong[1], Xinyuan Liu[1], Xingshu Chen[1], Shenghong Yang[2#], Yongjun Luo[1#]

(1 Department of Military Medical Geography, Army Health Service Training Base, Third Military Medical University(Army Medical University), Chongqing 40038, China; 2 Health Department of the 957th Hospital of PLA, Ali, Tibet, China 859000)

[#]Corresponding author:

Prof. Shenghong Yang, Health Department of the 957th Hospital of PLA, Ali, Tibet, 859000, China.

(E-mail: kevinysh0751@163.com)

Prof. Yongjun Luo, Army Health Service Training Base, Third Military Medical University(Army Medical University), Chongqing 400038, China.

(E-mail: luo.yongjun@qq.com)

Table S5 Detailed information of amplification for whole mtDNA genome

No	Primer sequences (5 '-3')	Regions	Annealing temperature(℃)	Product size (bp)
1	F:AACCAAACCCCAAAGACACC<br>R:GACTCTAGAATAGGATTGCGC	569-2941	60	2372
2	F:GTCCTAAACTACCAAACCTGC<br>R:GTGTTAGTCATGTTAGCTTG	2797-5193	60	2396
3	F:AGCAGTTCTACCGTACAACC<br>R: TTTGAAAAAGTCATGGAGGCC	5061-7497	60	2436
4	F：:GATTTGAGAAGCCTTCGCTTC<br>R: GCCAATAATGACGTGAAGTCC	7336-9819	60	2483
5	F:TCCCACTCCTAAACACATCC<br>R: AAACCCGGTAATGATGTCGG	9611-12111	60	2500
6	F:GCCCACGGGCTTACATC<br>R: GATTGTTAGCGGTGTGGTCG	11727-14159	60	2432
7	F:AATCTCCACCTCCATCATCACC<br>R: ACTGGTTGTCCTCCGATTCAGG	14046-15774	60	1728
8	F:TTCGCCTACACAATTCTCCG<br>R: TTTATGGGGTGATGTGAGCC	15591-626	60	1604

PCR was performed with a total reaction volume of 20 μl containing 10-50 ng of genomic DNA, 2.0 μl of 10×PCR mix (Dongsheng Bio Inc., Guangzhou, China), 1 μl of each primer (10mM), and 7 μl of ultrapure water. The PCR was carried out in a PTC-200 DNA Engine thermal cycler (Bio-Rad, Hercules, CA, USA) with a protocol of 94°C for 3 minutes; 35 cycles of 30 seconds at 94°C, 30 seconds at 60°C and 3 minutes at 72°C; and a final hold at 72°C for 10 minutes.
