# Supplementary material for: Mitochondrial DNA genomes revealed different patterns of high-altitude adaptation in high-altitude Tajiks compared with Tibetans and Sherpas
Source: Sci Rep. 2020 Jun 29;10:10592. doi: 10.1038/s41598-020-67519-z (PMC7324373; doi:10.1038/s41598-020-67519-z)
Supplement: Supplementary file 6 — Supplementary Table 6. [file 41598_2020_67519_MOESM6_ESM.pdf]

Mitochondrial DNA genomes revealed different patterns of high-altitude adaptation in high-altitude Tajiks compared with Tibetans and Sherpas

Yu Chen[1], Liang Gong[1], Xinyuan Liu[1], Xingshu Chen[1], Shenghong Yang[2#], Yongjun Luo[1#]

(1 Department of Military Medical Geography, Army Health Service Training Base, Third Military Medical University(Army Medical University), Chongqing 40038, China; 2 Health Department of the 957th Hospital of PLA, Ali, Tibet, China 859000)

[#]Corresponding author:

Prof. Shenghong Yang, Health Department of the 957th Hospital of PLA, Ali, Tibet, 859000, China.

(E-mail: kevinysh0751@163.com)

Prof. Yongjun Luo, Army Health Service Training Base, Third Military Medical University(Army Medical University), Chongqing 400038, China.

(E-mail: luo.yongjun@qq.com)

Table S6 Sequencing primers of whole mtDNA genome

No	5 ' position	Primer sequence(5'-3')
1	569	F:AACCAAACCCCAAAGACACC
2	626	F:TTTATGGGGTGATGTGAGCC
3	1305	F:GTAAGCGCAAGTACCCACG
4	2070	F:AATTTGCCCACAGAACCCTC
5	2797	F:GTCCTAAACTACCAAACCTGC
6	3568	F:CGCTCTTCTACTATGAACCC
7	4322	F:ATAATAGGAGCTTAAACCCCC
8	5061	F:AGCAGTTCTACCGTACAACC
9	5828	F:GAAAATCACCTCGGAGCTGG
10	6563	F:ACCTCAACACCACCTTCTTC
11	7336	F:GATTTGAGAAGCCTTCGCTTC
12	8080	F:TCTTGCACTCATGAGCTGTC
13	8815	F:CTCATTTACACCAACCACCC
14	9611	F:TCCCACTCCTAAACACATCC
15	10380	F:TCTGGCCTATGAGTGACTAC
16	11107	R:TTCACAGCCACAGAACTAATC
17	11381	R:AAGTGGAGTCCGTAAAGAGG
18	12862	R:AAACCGATATCGCCGATACG
19	13627	F:AAGCGAGGTTGACCTGTTAG
20	14046	R:AATCTCCACCTCCATCATCACC
21	15774	F:ACTGGTTGTCCTCCGATTCAGG
22	15591	R:TTCGCCTACACAATTCTCCG
